# Synthesis of Aggregation-Induced Emission-Active Conjugated Polymers Composed of Group 13 Diiminate Complexes with Tunable Energy Levels via Alteration of Central Element

**DOI:** 10.3390/polym9020068

**Published:** 2017-02-16

**Authors:** Shunichiro Ito, Amane Hirose, Madoka Yamaguchi, Kazuo Tanaka, Yoshiki Chujo

**Affiliations:** Department of Polymer Chemistry, Graduate School of Engineering, Kyoto University, Katsura, Nishikyo-ku, Kyoto 615-8510, Japan; shunito@chujo.synchem.kyoto-u.ac.jp (S.I.); amanehirose@yahoo.co.jp (A.H.); amanehirose@yahoo.co.jp (M.Y.)

**Keywords:** aggregation-induced emission, conjugated polymer, boron, gallium

## Abstract

Conjugated polymers containing boron and gallium diiminate complexes were prepared with various electron-donating comonomers via pre- and post-complexation methods, respectively. From a comparison of emission quantum yields between solution and film states, it was seen that all polymers containing group 13 elements possessed an aggregation-induced emission property. Additionally, the frontier orbital energies and the optical and electrochemical properties of the polymers can be tuned by altering a central element at the complex moieties as well as by changing a comonomer unit. In particular, it was demonstrated that the gallium atom can contribute to stabilizing the energy levels of the lowest unoccupied molecular orbitals, resulting in narrow band gaps of the conjugated polymers. This study presents the potential of gallium not only for preparing solid-state emissive conjugated polymers but also for fabricating low-band gap materials by employing the conjugated ligand.

## 1. Introduction

The introduction of heteroatoms into conjugated polymers is one of the valid strategies for obtaining the unique properties originating from each element. Various types of functional materials have been developed based on these polymers and applied for modern opto-electronic devices such as organic light-emitting diodes [1,2], photovoltaic cells [3,4,5,6], and sensors [7,8]. We have recently proposed the concept of “element-block polymers” for material design based on heteroatom-containing polymers [9]. By combination with “element-blocks”, which are defined as a functional minimum unit composed of heteroatoms, a wide variety of functional polymers involving inorganic elements can be produced. For example, boron-containing polymers have attracted attention as an emissive “element-block” for luminescent materials [10]. Owing to the emissive properties and stability of boron-containing “element-blocks”, various kinds of boron-containing conjugated molecules including polymers possessing useful luminescent properties such as near-infrared emission [11,12], a sharp intense spectrum [13], light-harvesting antenae [14,15,16], and aggregation-induced emission (AIE) [17,18], which can be obtained only in the condensed state of luminescent dyes without aggregation-caused quenching (ACQ), have been reported [19,20,21,22]. These materials are expected to be a key material for realizing advanced optical devices.

By changing a type of heteroatom in the “element-block”, superior properties and functions were often induced. For instance, Seferos et al. have recently reported that the photophysical and/or electronic properties of conjugated polymers containing benzochalcogenodiazoles [23] or chalcogenophenes [24] were significantly dependent on the type of chalcogen atom. Heeney et al. presented a series of low-band gap polymers containing chalcogenopene [25] or heteroles [26] in the polymer main-chains and their carrier transport ability with high efficiency. Tomita et al. have established preparation of various types of chalcogenophenes [27] and heteroles [28] via the polymer reactions, utilizing titanacyclobutadiene-2,5-diyl units as an intermediate. In particular, useful properties for application to modern electronic devices have been obtained in these polymers.

Replacement of the central metal in functional luminescent boron-containing complexes with heavier group 13 elements induced drastic changes in the electronic structures of the complex [29]. Recently, it was demonstrated that the replacement of boron with gallium can enhance a solid-state emissive property with the AIE-active diiminate complex [30]. Owing to the larger size of gallium than boron, ACQ was efficiently suppressed even in the crystalline state. Then, larger emission intensity was obtained from the crystalline sample of the gallium complex. Furthermore, it was found that gallium can create channels in the crystal packing. As a result, vapochromic luminescence was detected in the presence of volatile organic molecules. So far, several types of gallium-containing conjugated polymers have been developed, and distinctive features were indeed obtained from some of these gallium-containing polymers [31,32,33,34,35]. However, systematic information on the differences in electronic properties between boron and gallium is still required for establishing material design based on the advantageous properties of gallium. Therefore, our next goal is to offer a comparison study of photophysical properties with conjugated polymers involving boron and gallium complexes for comprehending the peculiar functions originating from the intrinsic properties of each group 13 element.

Herein, a series of conjugated polymers composed of boron and gallium complexes with the diiminate structure, which promises to be a versatile scaffold for realizing AIE and encouraging electronic delocalization through polymer main-chains, was prepared by the pre- and post-complexation methods. Initially, as expected, boron- and gallium-containing conjugated polymers can work as an AIE-active material. Moreover, optical measurements with the synthesized polymers revealed that gallium should be responsible for narrowing the energy band gaps of the polymers. From the mechanical studies including theoretical approaches, it was proposed that stabilization of the energy levels of molecular orbitals should be induced not by the charge transfer process but by the intrinsic property of gallium. This is, to the best of our knowledge, the first study to experimentally present the feasibility of gallium not only for AIE-active conjugated polymers but also for low-band gap materials by employing the conjugated ligand.

## 2. Results and Discussion

### 2.1. Synthesis and Characterization of the Conjugated Copolymers

**Scheme 1:**
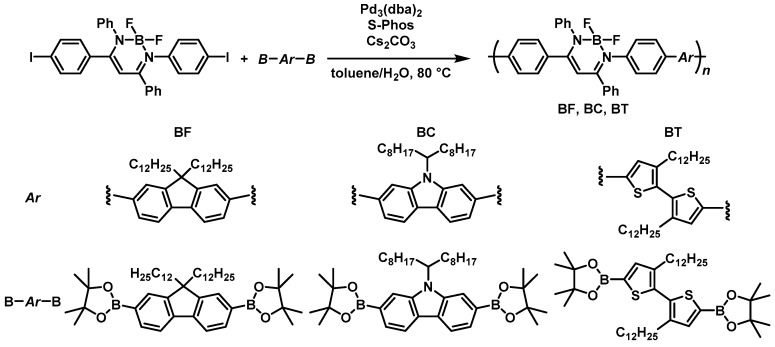
Synthetic schemes of the polymers containing boron diiminate.

The diiodo-substituted boron diiminate monomer **IB** was synthesized according to our previous report [36]. The conjugated copolymer **BF**, composed of boron diiminate and 9,9-didodecylfluorene, was prepared by the palladium-catalyzed Suzuki–Miyaura cross coupling polymerization with **IB** and [9,9-bis(dodecyl)-9H-fluorene-2,7-diyl]bisboronic acid in the presence of 2-dicyclohexylphosphino-2′,6′-dimethoxybiphenyl (S-Phos), tris(dibenzylideneacetone)dipalladium (Pd_2_(dba)_3_) and cesium carbonate in the toluene/water mixture solvent (Scheme 1). Because of high stability under the polymerization condition, a post-polymerization method was accomplished with boron diiminate. The other conjugated copolymers containing different types of comonomers (**BC** and **BT**) were also synthesized with the same post-polymerization method using the corresponding diboronic acid bis(pinacol) esters. The number- and weight-average molecular weights (*M*_n_ and *M*_w_), the molecular weight distribution (*M*_w_/*M*_n_) and the degree of polymerization (*n*) were estimated by size-exclusion chromatography (SEC) in chloroform as an eluent with polystyrene standards (Table 1). Their chemical structures were confirmed by comparing the ^1^H and ^13^C NMR spectra of the products with those of the monomer (Figures S1–S9). From these characterization data, it was concluded that a series of conjugated polymers involving boron diiminate were obtained.

**Scheme 2:**
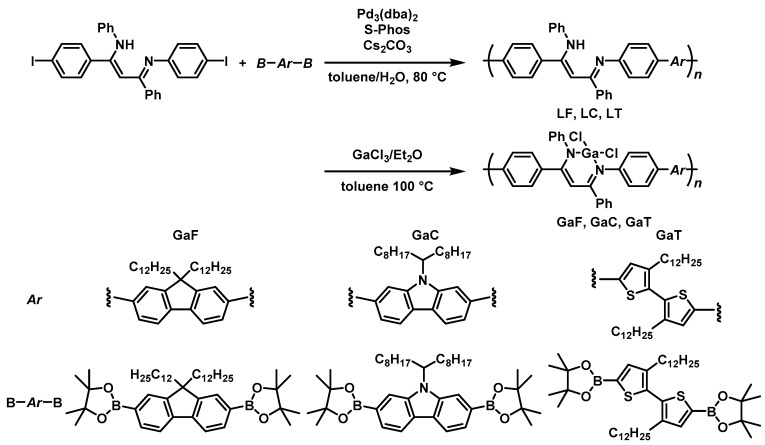
Synthetic schemes of the polymers containing gallium diiminate.

To synthesize the conjugated polymers composed of gallium diiminate, the diiodo-substituted gallium diiminate **IGa** was initially prepared and used instead of **IB** under the same polycondensation condition. The gallium diiminate monomer, however, was decomposed during the polymerization due to instability under the basic condition of the Suzuki–Miyaura coupling reaction. In the ^1^H NMR spectrum of the mixture, the peaks around 13 ppm assigned to the diimine ligand (N*H*) were observed after the reaction. This result obviously indicated that bond cleavage between gallium and nitrogen should occur during polymerization. Therefore, the post-complexation method after polymerization was performed for obtaining desired polymers by employing the polymeric ligands involving the diimine structure, which promises to make a stable complexation with various metal cations as well as group 13 elements [37,38,39,40]. The polymeric ligands (**LF**, **LC**, and **LT**) were synthesized via polycondensation with the diiodo-substituted ligand **IL** and each corresponding comonomer (Scheme 2). Then, the products were treated with gallium trichloride in toluene at 100 °C under argon atmosphere in the presence of triethylamine. According to the ^1^H NMR spectra of all polymers, it was observed that the peaks around 13 ppm originating from the polymeric ligands (N*H*) completely disappeared after the treatment (Figures S10–S12). The characteristic signals of the methine protons in the ligand moieties were shifted to the lower-field region after the treatment. Moreover, the repeating units of each polymer were detected in the MALDI-TOF-MS spectra (Figures S13–S15). Additionally, it was found that the vibrational peaks assigned to Ga–N bonds appeared in the FT-IR spectra of the products (Figure S16). Furthermore, a decrease in molecular weight was hardly observed in GSC analyses (Table 1). These data indicate that the post-complexation methods quantitatively proceeded without undesired side reactions or chain scission. From the results of the SEC analyses, the physical properties of the products were determined, and it was confirmed that polymeric products were obtained. The resulting polymers containing gallium diiminate had enough stability for evaluating thermal characteristics as well as optical properties (Figures S17–S19). From these results, including those from boron diiminate polymers, it was concluded that a series of conjugated polymers containing boron and gallium can be obtained with enough length for evaluating the influence of the group 13 elements on electronic structures of polymer main-chains. All polymers showed high solubility in common organic solvents such as chloroform, dichloromethane, and tetrahydrofuran (THF), and good film formability (Figure S21). Homogeneous amorphous films were readily obtained with the spin-coating methods.

### 2.2. Optical Properties

**Figure 1:**
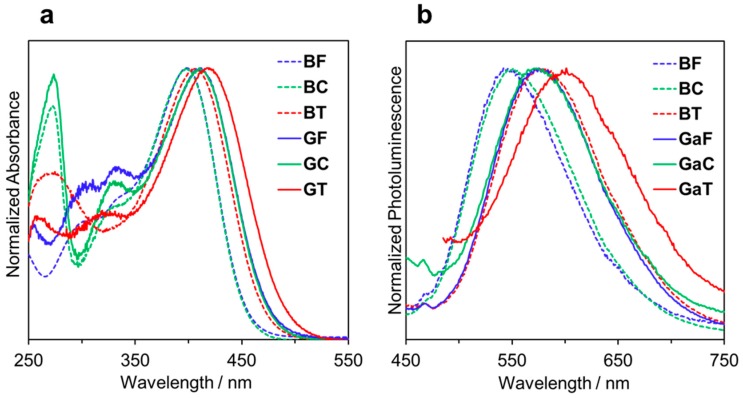
(**a**) Normalized UV–vis absorption spectra of the polymers in CHCl_3_ (1.0 × 10^−5^ M); (**b**) normalized photoluminescence spectra of the polymers in the spin-coated thin-film state.

To evaluate electronic properties in the ground state, the UV-vis absorption spectra were measured with the chloroform solutions of the polymers (1.0 × 10^−5^ M based on the repeat unit), as shown in Figure 1a. The results are listed in Table 2. All compounds exhibited strong absorption bands in the longer wavelength region by about 30 nm than those of monomeric complexes [30,36]. This means that electronic delocalization should occur through polymer main-chains. The absorption bands of the polymers containing the bithiophene unit (**BT** and **GaT**) showed larger bathochromic shifts by 5–10 nm compared to those of polymers composed of fluorene (**BF** and **GaF**) and carbazole (**BC** and **GaC**). These results can be explained by the order of strength of the electron-donating ability of the comonomer units. The most significant point was the fact that the polymers containing the gallium complex exhibited red-shifted absorption spectra compared to those from boron-containing polymers with corresponding comonomers. In particular, according to the optical band gaps (*E*_g_^opt^) of the synthesized polymers determined by the onset wavelength of the absorption bands in the visible region, it was clearly indicated that the optical band gaps of these polymers were significantly narrowed by the alteration of the central element on the diiminate units from boron to gallium (Table 3). 

The photoluminescence properties of the polymers were evaluated to investigate electronic properties in the excited state with the diluted solutions (1.0 × 10^−5^ M) and the spin-coated thin films. All polymers exhibited slight emission in the solution state, and the absolute photoluminescence quantum yields were determined to be below 0.01 with the integrating sphere method with the solutions of all polymers (Table 2). In contrast, relatively larger quantum yields were obtained from thin-film samples than from solutions. The photoluminescence quantum yields in the film state (Φ_PL_^film^) were determined to be about 0.05 (Figure 1b). These data indicate that the polymers possess an AIE property, similarly to the diiminate complex moieties [30,36]. Compared to the degree of emission enhancement of the monomers in the aggregation state, a relatively smaller extent of emission enhancement was observed from the polymers [30,36]. According to previous works, the AIE-active alternating conjugated copolymers having high planarity through polymer main-chains also showed similar emission efficiencies in the film state [22,36,41]. Inter-chain interaction such as π–π stacking, especially at the comonomer units, should suppress AIE in the condensed state. It should be remarked that the emission maxima of these polymers were also shifted to the longer wavelength region in the same order of their absorption spectra in the solutions. This trend was comparable to the order of the values of *E*_g_^opt^. To comprehend the mechanism for the narrowing effect on the band gap by gallium, further experiments were performed. 

### 2.3. Contribution from Electronic Interaction

**Figure 2:**
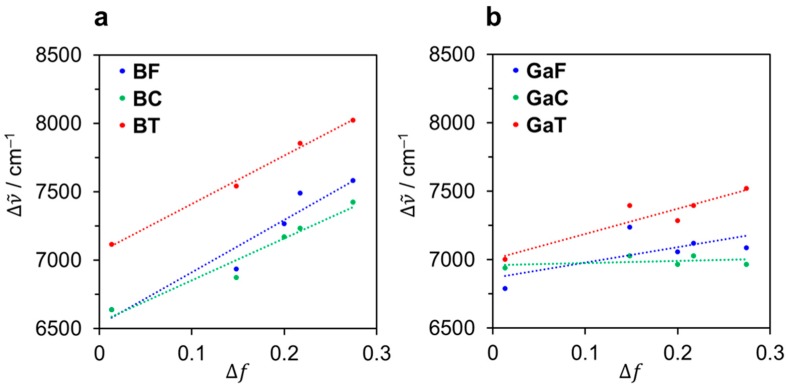
Lippert–Mataga plots for (**a**) boron and (**b**) gallium polymers. Solvents and Δ*f*: toluene, 0.0132; chloroform, 0.148; ethyl acetate, 0.200; dichloromethane, 0.217; dimethylformamide, 0.274.

Narrow energy gaps can often be observed in conjugated polymers an having intramolecular charge transfer (ICT) property that originated from a strong donor–acceptor electronic interaction through polymer main-chains [37,38]. In particular, by enhancing electron-donating and/or accepting abilities, a further narrowing effect can be induced. Initially, to examine the ICT characters of the polymers, Lippert–Mataga plots [39,42,43,44,45] were prepared according to the empirical formula (see Supplementary Materials, Tables S1–S6, Figures S22 and S23). The degree of ICT can be estimated from the slope of a fitting line (Figure 2, Table S7). In addition, the dipole moments in the excited state were approximately calculated with hypothetical Onsager radii and estimated dipole moments in the ground state [42,43,44,45]. Although all polymers including boron and gallium showed ICT characters, contrary to the presumption that larger ICT character should be observed from the gallium-containing polymers, the weak dependency of the Stokes shift (Δ$\tilde{\nu }$) values was observed on a solvent polarity (Lippert polarity parameter; Δ*f*) relative to the boron-containing polymers (boron: 3070–3830 cm^−1^; gallium: 148–1840 cm^−1^). The smaller differences in the dipole moment between ground and excited states were correspondingly obtained from the gallium-containing polymers (boron: 10.9–12.6 D; gallium: 2.61–8.69). These data mean that ICT characters of the gallium-containing polymers should be weaker than those of boron. Since the electronegativity of gallium (1.81) is smaller than that of boron (2.04), it is proposed that the relatively smaller electron-accepting property of the diiminate unit might be obtained in the gallium-containing polymers [40,46].

### 2.4. Electrochemical Properties of the Polymers

Electrochemical characteristics were collected via cyclic voltammetry (CV) to gather further information on the electronic properties of the synthesized polymers (Figure S20). The conditions of the CV measurement are disclosed in detail in the Supplementary Materials. Accordingly, in the reduced wave in the CV spectra, reversible redox processes were obtained, especially from the boron-containing polymers. Even at the third reduction attributable to the reduction at the diiminate moieties, clear reversible processes were detectable. It should be mentioned that critical precipitation or insoluble coating were hardly generated on the surface of electrodes after scanning. These data mean that electron injection followed by removal proceeded smoothly. In modern organic light-emitting diodes, the use of emitting layers having charge carrier ability is advantageous, and luminescent materials with charge transfer characters are attractive candidates to meet demand [47,48]. Owing to the bright solid-state emission and potential electron-carrier ability of group 13 element-containing complexes, our materials might be applicable in modern opto-electronic devices. The energy levels of a highest occupied molecular orbital (HOMO) and a lowest unoccupied molecular orbital (LUMO) were estimated with data from a CV analysis and a UV–vis absorption measurement (Table 3) [49,50,51]. The commonly utilized scale of −4.8 eV was employed as the absolute energy level of Fc/Fc^+^ according to previous reports presenting the correction protocol for CV-derived redox potentials to energy levels of frontier orbitals in the materials [49,50,51]. It was shown that the LUMO levels of the polymers containing gallium were significantly lower than those of the boron-containing polymers in comparison with polymers having the same comonomer units, whereas the HOMO levels were similar. This fact means that stabilizations of the LUMO levels by gallium should play critical roles in narrowing the HOMO–LUMO energy gaps. From these results, it is concluded that a narrowing of the optical band gaps should be derived from the stabilization of the LUMO levels by gallium. 

### 2.5. Density Functional Theory Calculations

**Figure 3:**
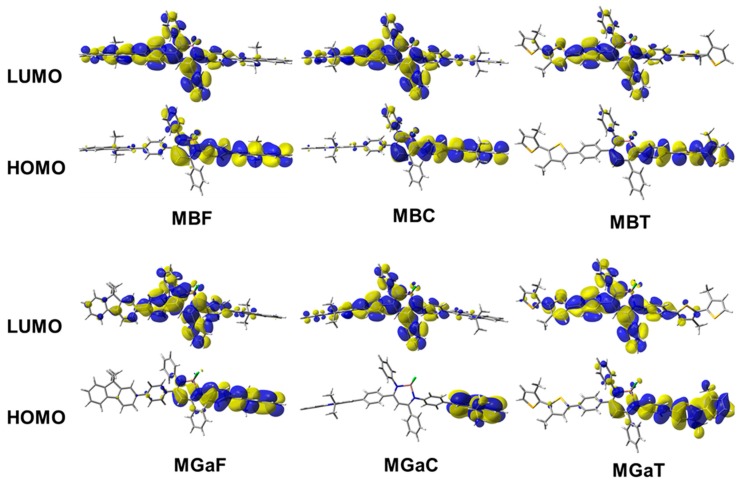
Frontier orbital distributions of the model compounds obtained from the DFT calculations.

To complement information on the electronic structures of the polymers, quantum calculation was carried out with the density functional theory (DFT) and time-dependent DFT (TD-DFT) methods with the model compounds for the polymeric compounds at the B3LYP level of theory with 6-31+G(d,p) basis set using the Gaussian 09 program package (Figure S24) [52]. The model compounds **MXF**, **MXC**, and **MXT** (**X** = **B** or **Ga**) were composed of comonomer–complex–comonomer compositions and have methyl groups instead of long alkyl chains for simplification. The calculated molecular orbital distributions are shown in Figure 3. The results indicated that the HOMOs and LUMOs were mainly located at the comonomer unit and the diiminate complex moiety in all compounds, respectively. It should be emphasized that the LUMOs of the gallium compounds were obtained at a significantly lower energy region than those of the boron ones. In contrast, the HOMO levels of the gallium compounds were comparable (Table S8). These results apparently corresponded with the experimental data from the spectroscopic analyses. In this study, relatively electron-donating comonomers, which are intrinsically able to lift the energy levels of molecular orbitals, were conjugated with the complexes. From the results of calculations, it was shown that HOMOs of the polymers were mainly localized at the comonomer units. From these results, it is proposed that the electronic properties of the comonomer units are responsible for the energy levels of HOMO in the polymers. Thus, comparable values should be obtained. Furthermore, electronic delocalization at the boron complex moieties was observed in HOMOs, while clear localization at the comonomer units was presented in the gallium compounds. These results imply that a larger degree of ICT characters might be observed from boron-containing polymers compared to gallium because of the larger overlap of molecular orbitals.

The TD-DFT calculations revealed that the absorption bands of the model compounds at the lowest energy region should be attributed to the transitions between the frontier orbitals (Table S9). From these data, it was strongly suggested that the red shifts observed in the absorption and photoluminescence spectra should be derived from the enhanced stabilization effect of the LUMO levels by the alteration of the central element from boron to gallium. Because the LUMOs can be localized at the diiminate complex moieties, stabilization should occur efficiently, leading to narrow band gaps. The complex with the diiminate ligand could be favorable for modulating main-chain conjugation in conjugated polymers. The influence of the introduction of the group 13 element into the ligand and the type of halogen on the gallium atom was also evaluated by the DFT calculation (Figure S25). In summary, it was clearly indicated that boron and gallium atoms can contribute to lowering the LUMO level of the ligand. Interestingly, the tendency of stabilization of the LUMOs was independent of the kind of the halogen atoms on the gallium atom. This fact also supports the idea that stabilization of the LUMO energy could be induced owing to the intrinsic property of gallium. Weak interaction of the 3d orbital of gallium with LUMOs was observed for **MGa**, **MGaF**, **MGaF**, **MGaC**, and **MGaT** (Figure S25). Then, energy levels of LUMOs might be lowered.

## 3. Conclusions

The conjugated polymers containing boron and gallium diiminates were synthesized with various electron-donating comonomers via pre- and post-complexation methods, respectively. From the series of optical measurements, it was observed that all polymers containing group 13 elements exhibited an AIE property. Moreover, it was shown that the frontier orbital energies and the optical and electrochemical properties of the polymers were easily tuned by altering a central element at the complex moieties as well as by changing a comonomer unit. Group 13 element-containing polymers often showed narrow band-gaps originated from low LUMO levels because of the electron-deficient nature of elements. In this study, it was clearly demonstrated that the diiminate polymers are also an effective scaffold for expressing elemental characteristics from conjugated polymers. In particular, it was found that the gallium atom can be responsible for stabilizing the LUMO levels of the polymers. This means that the replacement of gallium in the diiminate complexes could be a promising strategy for obtaining low-lying emissive materials without a loss of luminescent properties via the heavy atom effect. Furthermore, it was implied that the polymeric ligands that are applicable in the post-complexation method might be a versatile scaffold for constructing AIE-active conjugated polymers by employing various kinds of metals. Our findings should be useful for designing advanced polymeric materials possessing superior opto-electronic properties.

## Figures and Tables

**Table 1:** Polymer properties of the products ^a^.

Polymer	*M*_n_	*M*_w_	*M*_w_/*M*_n_	*n* ^b^
BF	13,500	34,000	2.5	15
BC	15,200	38,000	2.5	13
BT	12,200	28,800	2.4	18
LF	9200	27,000	2.9	11
LC	7000	15,400	2.2	9.0
LT	7100	14,000	2.0	8.1
GaF	9400	18,000	1.9	9.4
GaC	10,000	18,000	1.8	11
GaT	8800	14,000	1.6	8.7

^a^ Estimated by SEC with the polystyrene standards in CHCl_3_; ^b^ Average number of repeating units calculated from *M*_n_ and molecular weights of the repeating units.

**Table 2:** Photophysical properties of the synthesized polymers.

Polymer	λ_abs_^solution^/nm	λ_abs_^film^/nm	ε × 10^4^/M^−1^·cm^−1^ ^a^	λ_PL_^solution^/nm ^b^	λ_PL_^film^/nm ^c^	Φ_PL_^film c,d^
BF	399	399	5.99	545	545	0.07
BC	397	404	4.00	545	552	0.07
BT	404	417	4.96	581	575	0.07
GF	411	415	3.94	571	575	0.05
GC	410	414	3.91	576	573	0.05
GT	420	424	3.83	610	601	0.03

^a^ Molar absorption coefficients of the absorption maxima in the longer wavelength region; ^b^ Excited at λ_abs_^solution^; ^c^ Excited at λ_abs_^film^; ^d^ Determined with the integrated sphere method.

**Table 3:** Results of the absorption spectroscopy and the cyclic voltammetry.

Polymer	λ_onset_/nm ^a^	*E*_g_^opt^/eV ^b^	*E*_red_^CV^/V ^c,d^	*E*_HOMO_/eV ^e^	*E*_LUMO_/eV ^f^
BF	451	2.74	−1.27	−6.27	−3.53
BC	452	2.74	−1.36	−6.18	−3.44
BT	467	2.65	−1.36	−6.10	−3.45
GaF	479	2.59	−1.20	−6.19	−3.60
GaC	475	2.61	−1.26	−6.15	−3.54
GaT	488	2.54	−1.26	−6.08	−3.54

^a^ Onset value of the UV-vis spectra measured in THF (1.0 ×10^−5^ M); ^b^ Optical band gap estimated from the onset wavelength of the UV–vis spectra in THF; ^c^ Cyclic voltammetry was carried out in THF with 0.1 M tetrabutylammonium perchlorate as a supporting electrolyte and the Fc/Fc^+^ redox couple as an external standard; ^d^ *E*_red_ is the onset potential of first reduction wave; ^e^ Calculated from LUMO and optical band gap (*E*_g_^opt^) of the synthesized compounds, *E*_HOMO_/eV = *E*_LUMO_/eV − *E*_g_^opt^/eV; ^f^ Calculated from the empirical formula, *E*_LUMO_/eV = −*E*_red_/eV − 4.80.

## Supplementary Materials

The following are available online at www.mdpi.com/2073-4360/9/2/68/s1. Experimental protocols and characterization data, Scheme S1: preparation of gallium polymers, Figures S1–S12: NMR spectra, Figures S13–S15, Figure S16: IR spectra, Figure S17: XRF spectra, Figure S18: TGA profiles, Figure S19 DSC profiles, Figure S20: cyclic voltammograms, Figures S21–S23: normalized UV−vis absorption spectra, Figure S24: DFT calculation results, Figure S25: energy diagrams and molecular orbitals, Tables S1–S7: optical data for preparing the Lippert−Mataga plots, Table S8: energy levels, Table S9: assignment to electron transition.
