# Supplementary material for: Synthesis of Aggregation-Induced Emission-Active Conjugated Polymers Composed of Group 13 Diiminate Complexes with Tunable Energy Levels via Alteration of Central Element
Source: Polymers (Basel). 2017 Feb 16;9(2):68. doi: 10.3390/polym9020068 (PMC6432313; doi:10.3390/polym9020068)
Supplement: Supplementary file 1 [file polymers-09-00068-s001.pdf]

Supporting Information

Synthesis of Aggregation-Induced Emission-Active Conjugated Polymers Composed of Group 13 Diiminate Complexes with Tunable Energy Levels via Alteration of Central Element

Shunichiro Ito, Amane Hirose, Madoka Yamaguchi, Kazuo Tanaka and Yoshiki Chujo**

Department of Polymer Chemistry, Graduate School of Engineering, Kyoto University, Katsura, Nishikyo-ku, Kyoto 615-8510, Japan

Corresponding author E-mail: kazuo123@chujo.synchem.kyoto-u.ac.jp; chujo@chujo.synchem.kyoto-u.ac.jp

Tel: (+81)-75-383-2604. Fax: (+81)-75-383-2605

Experimental Section

Measurements: ^{1}H (400 MHz), ^{13}C (100 MHz) and 11B (128 MHz) NMR spectra were recorded on a JEOL JNM-EX400 spectrometer. In ^{1}H and ^{13}C NMR spectra, tetramethylsilane (TMS) was used as an internal standard in $CDCl_3$. ^{11}B NMR spectra were referenced externally with $BF_3{\cdot}OEt_2$ in $CDCl_3$. Analytical thin-layer chromatography (TLC) was performed with silica gel 60 Merck F254 plates. Column chromatography was performed with a Wakogel C-300 silica gel. Number-average molecular weight (M_n) and molecular weight distribution (M_w/M_n) values of all polymers were estimated by size exclusion chromatography (SEC) with a TOSOH 8020 series [a dual pump system (DP-8020), a column oven (CO-8020), and a degasser (SD-8020)] equipped with three consecutive polystyrene gel columns [TOSOH TSKgel: G2000H, G3000H and G4000H] and refractive-index (RI-8020) and ultraviolet detectors (UV-8020) at 40 °C. The system was operated at a flow rate of 1.0 mL/min with $CHCl_3$ as an eluent. Polystyrene standards were employed for calibration. UV−vis absorption spectra were recorded on a SHIMADZU UV-3600 spectrophotometer. Fluorescence emission spectra were measured with a HORIBA JOBIN YVON Fluoromax-4P spectrofluorometer, and photoluminescence quantum yields were calculated as an absolute value by the integrating sphere method. Thermogravimetric analysis (TGA) was performed on an EXSTAR TG/DTA6220, Seiko Instrument, Inc. with the heating rate of 10 °C/min up from 40 to 500 °C under nitrogen flowing (200 mL/min). The decomposition temperatures (T_{5d}) were determined from the temperature of the 5% weight loss. DSC thermograms were carried out on a SII DSC 6220, Seiko Instrument, Inc. The sample on the sealing aluminum pan was heated at the rate of 10 °C/min under nitrogen flowing (20 mL/min). Cyclic voltammetry (CV) was carried out on a BAS ALS-Electrochemical-Analyzer Model 600D under an argon atmosphere. A three-electrode cell equipped with a glassy carbon (GC) working electrode, a Pt wire counter electrode, and an Ag/Ag^+ reference electrode. The measurements were performed in degassed tetrahydrofuran with tetrabutylammonium perchlorate (0.1 M) as the supporting electrolyte at a scan rate of 50 mVs^{-1}. The absolure energy level of ferrocene/ferrocenium (Fc/Fc^+) which utilized as the external standard was set to be 4.8 eV below vacuum. Ferrocene (Aldrich Chemical, Co.) was used as received. The LUMO level was calculated from the

equation: E_{LUMO} / eV = – E_{red} / eV – 4.8, and the HOMO level was calculated from: E_{HOMO} / eV = E_{LUMO} / eV – E_g^{opt} / eV. X-ray fluorescence analysis (XRF) of gallium content in the samples was carried out on a Rigaku XRF-Primini spectrometer. FT-IR spectra were obtained on a Shimadzu IRPrestige-21 spectrometer.

Materials: All reactions were performed under argon atmosphere. $BF_3 \cdot OEt_2$ (Sigma-Aldrich Co., LLC.; SA), gallium chloride anhydrous (Tokyo Chemical Industry Co., Ltd.; TCI), deoxidized toluene (Wako Pure Chemical Industries, Ltd.; Wako), [9,9-bis(dodecyl)-9H-fluorene-2,7-diyl]bisboronic acid (SA), 9-(9-heptadecanyl)-2,7-bis(4,4,5,5-tetramethyl-1,3,2-dioxaborolan-2-yl)carbazole (TCI), 2-dicyclohexylphosphino-2',6'-dimethoxybiphenyl (S-Phos, Wako), tris(dibenzylideneacetone)dipalladium ($Pd_2(dba)_3$, TCI), and cesium carbonate (Wako) were used as received. Diethylether and triethylamine (Wako Pure Chemical Industries, Ltd.) were purified using a two-column solid-state purification system (Glasscontour System, Joerg Meyer, Irvine, CA). **IB**, **IL**, and 2,2'-(3,3'-didodecyl-[2,2'-bithiophene]-5,5'-diyl)bis(4,4,5,5-tetramethyl-1,3,2-dioxaborolane) were prepared according to the literatures.[1,2]

Synthesis of BF: Water (0.5 mL) was added to the solution of **IB** (50 mg, 0.074 mmol), 9,9-didodecylfluorene-2,7-diboronic acid (44 mg, 0.074 mmol), $Pd_2(dba)_3$ (2.0 mg, 2.2 μmol), S-Phos (2.1 mg, 5.2 μmol) and cesium carbonate (241 mg, 74 mmol) in toluene (0.5 mL). The reaction mixture was stirred at 80 °C for 24 h under argon atmosphere, and then 1,4-dimethyl-2-iodobenzene (26 mg, 13 μL, 0.16 mmol) was added to the reaction mixture. The reaction mixture was stirred at 80 °C for 12 h. After cooling to room temperature, the reaction mixture was poured into a large amount of methanol to collect the polymer as a precipitate. After the filtration, the product was washed with ethanol and water. The precipitate was dissolved in a small amount of THF, and then the product was reprecipitated from methanol. The polymer collected by filtration was dried in vacuum to give **BF** a yellow solid in 81% yield. M_n = 13,500, M_w/M_n = 2.5. ^{1}H NMR ($CDCl_3$): δ = 7.73–7.07 (24H, Ar-H), 5.73 (1H, -CH=), 1.98 (4H, -CH_2-), 1.25–1.03 (36H, -C_7H_{15}), 0.88–0.84 (6H, -CH_3), 0.64 (4H, -CH_2-) ppm. ^{13}C NMR ($CDCl_3$): δ = 164.14, 151.84, 151.67, 142.69, 142.56, 141.90, 140.92, 140.73, 140.48, 139.45, 136.59, 135.02, 129.66,

129.17, 128.27, 128.03, 127.87, 126.97, 126.71, 125.95, 122.61, 122.39, 121.32, 119.97, 99.88, 55.29, 40.38, 31.88, 30.03, 29.56, 29.50, 29.28, 23.87, 22.65, 14.06 ppm. ^{11}B NMR ($CDCl_3$): δ = 1.76 ppm.

Synthesis of BC: Water (2 mL) was added to the solution of **IB** (0.10 g, 0.15 mmol), 9-(9-heptadecanyl)-2,7-bis(4,4,5,5-tetramethyl-1,3,2-dioxaborolan-2-yl)carbazole (0.098 g, 0.15 mmol), $Pd_2(dba)_3$ (1.4 mg, 1.5 μmol), S-Phos (2.4 mg, 5.9 μmol) and cesium carbonate (0.48 g, 1.5 mmol) in toluene (3.0 mL). The reaction mixture was stirred at 80 °C for 24 h under argon atmosphere, and then 1,4-dimethyl-2-iodobenzene (50 mg, 25 μL, 0.20 mmol) was added to the reaction mixture. The reaction mixture was stirred at 80 °C for 14 h. After cooling to room temperature, the reaction mixture was poured into a large amount of methanol to collect the polymer as a precipitate. After the filtration, the product was washed with ethanol and water. The precipitate was dissolved in a small amount of THF, and then the product was reprecipitated from methanol. The polymer collected by filtration was dried in vacuum to give **BC** as a yellow solid (91%). M_n = 12,200, M_w/M_n = 2.4. ^{1}H NMR ($CDCl_3$): δ = 8.11–7.14 (24H, Ar-*H*), 5.77 (1H, -C*H*=), 4.61 (1H), 2.30 (2H), 1.94 (2H), 1.40–0.78 (30H) ppm. ^{13}C NMR ($CDCl_3$): δ = 164.17, 143.07, 141.91, 136.60, 135.00, 132.62, 129.71, 129.17, 128.25, 127.42, 127.10, 126.33, 122.73, 120.39, 118.31, 99.89, 33.81, 31.69, 29.36, 29.26, 29.10, 27.93, 26.81, 24.92, 22.53, 22.42, 13.99 ppm. ^{11}B NMR ($CDCl_3$): δ = 1.86 ppm.

Synthesis of BT: Water (2 mL) was added to the solution of **IB** (0.12 g, 0.16 mmol), 3,3'-didodecyl-2,2'-bithiophene-5,5'-diboronic acid bis(pinacol) ester (0.11 g, 0.16 mmol), $Pd_2(dba)_3$ (1.5 mg, 1.6 μmol), S-Phos (2.6 mg, 6.4 mmol) and cesium carbonate (0.52 g, 1.6 mmol) in toluene (2.5 mL). The reaction mixture was stirred at 80 °C for 24 h under argon atmosphere, and then 1,4-dimethyl-2-iodobenzene (50 mg, 25 μL, 0.20 mmol) was added to the reaction mixture. The reaction mixture was stirred at 80 °C for 12 h. After cooling to room temperature, the reaction mixture was poured into a large amount of methanol to collect the polymer as a precipitate. After the filtration, the product was washed with ethanol and water. The precipitate was dissolved in a small amount of THF, and then the product was reprecipitated from methanol. The polymer collected by filtration was dried in vacuum to give **BT** as an orange solid (91%). M_n = 15,200, M_w/M_n = 2.5. ^{1}H NMR ($CDCl_3$): δ = 7.46–6.96 (20H, Ar-*H*), 5.68 (1H, -C*H*=), 2.50 (4H),

1.22–0.86 (46H) ppm. ^{13}C NMR ($CDCl_3$): δ = 164.11, 163.82, 143.65, 142.52, 142.24, 141.77, 141.13, 136.42, 135.23, 132.07, 129.77, 129.58, 129.09, 128.41, 128.26, 128.12, 127.74, 126.40, 125.58, 125.31, 124.92, 99.73, 31.89, 30.65, 29.64, 26.61, 29.53, 29.46, 29.42, 29.32, 29.08, 22.66, 14.08 ppm. ^{11}B NMR ($CDCl_3$): δ = 1.76 ppm.

Synthesis of LF: Water (10 mL) was added to the solution of **IL** (0.30 g, 0.48 mmol), 9,9-didodecylfluorene-2,7-diboronic acid (0.28 g, 0.48 mmol), $Pd_2(dba)_3$ (4.4 mg, 4.8 mmol), S-Phos (7.8 mg, 19 mmol) and cesium carbonate (1.6 g, 4.8 mmol) in toluene (5.0 mL). The reaction mixture was stirred at 80 °C for 24 h under argon atmosphere, and then 1,4-dimethyl-2-iodobenzene (40 mg, 20 μL, 0.16 mmol) was added to the reaction mixture. The reaction mixture was stirred at 80 °C for 15h. After cooling to room temperature, the reaction mixture was poured into a large amount of methanol to collect the polymer as a precipitate. After the filtration, the product was washed with ethanol and water. The precipitate was dissolved in a small amount of THF, and then the product was reprecipitated from methanol. The polymer collected by filtration was dried in vacuum to give **LF** as a brown solid (0.32 g, 78%).

LF M_n = 9,200, M_w/M_n = 2.9. ^{1}H NMR ($CDCl_3$): δ = 13.13 (1H, -N*H*-) 7.76–6.85 (24H, Ar-*H*), 5.49 (1H, -C*H*=), 1.99 (3H), 1.25–0.67 (47H) ppm.

Synthesis of LC: Water (10 mL) was added to the solution of **IL** (0.30 g, 0.48 mmol), 9-(9-heptadecanyl)-2,7-bis(4,4,5,5-tetramethyl-1,3,2-dioxaborolan-2-yl)carbazole (0.32 g, 0.48 mmol), $Pd_2(dba)_3$ (4.4 mg, 4.8 mmol), S-Phos (7.8 mg, 19 mmol) and cesium carbonate (1.6 g, 4.8 mmol) in toluene (5.0 mL). The reaction mixture was stirred at 80 °C for 24 h under argon atmosphere, and then 1,4-dimethyl-2-iodobenzene (40 mg, 20 μL, 0.16 mmol) was added to the reaction mixture. The reaction mixture was stirred at 80 °C for 15h. After cooling to room temperature, the reaction mixture was poured into a large amount of methanol to collect the polymer as a precipitate. After the filtration, the product was washed with ethanol and water. The precipitate was dissolved in a small amount of THF, and then the product

was reprecipitated from methanol. The polymer collected by filtration was dried in vacuum to give **LC** as an orange solid (0.35 g, 95%).

LC M_n = 7,000, M_w/M_n = 2.2. ^{1}H NMR ($CDCl_3$): δ = 8.12–6.89 (24H, Ar-*H*), 5.52 (1H, -C*H*=), 4.65 (1H), 2.51 (4H), 2.36 (2H), 1.96 (2H), 1.18–0.78 (30H) ppm.

Synthesis of LT: Water (15 mL) was added to the solution of **IL** (0.30 g, 0.48 mmol), 3,3’-didodecyl-2,2’-bithiophene-5,5’-diboronic acid bis(pinacol) ester (0.36 g, 0.48 mmol), $Pd_2(dba)_3$ (4.4 mg, 4.8 mmol), S-Phos (7.8 mg, 19 mmol) and cesium carbonate (1.6 g, 4.8 mmol) in toluene (6.0 mL). The reaction mixture was stirred at 80 °C for 24 h under argon atmosphere, and then 1,4-dimethyl-2-iodobenzene (40 mg, 20 μL, 0.16 mmol) was added to the reaction mixture. The reaction mixture was stirred at 80 °C for 17 h. After cooling to room temperature, the reaction mixture was poured into a large amount of methanol to collect the polymer as a precipitate. After the filtration, the product was washed with ethanol and water. The precipitate was dissolved in a small amount of THF, and then the product was reprecipitated from methanol. The polymer collected by filtration was dried in vacuum to give **LT** as a brown solid (0.33 g, 79%).

LT M_n = 7,000, M_w/M_n = 2.0. ^{1}H NMR ($CDCl_3$): δ = 13.07 (1H, -N*H*-) 7.51–6.73 (20H, Ar-*H*), 5.43 (1H, -C*H*=), 2.51 (4H), 1.23–0.85 (46H) ppm.

Synthesis of GF, GC and GT: $GaCl_3$ (15 eq.) in diethyl ether (10 mL) was added to the solution of the ligand polymer in toluene (30 mL) at room temperature under argon atmosphere. NEt_3 (15 eq.) was added to the mixture solution. The mixture solution was stirred at 100 °C for 15 h. After the solvent was removed by a rotary evaporator, the product was extracted by $CHCl_2$ (10 mL). The filtrate was dropped into methanol (50 mL), leading to precipitating the desire compound. The solid collected by filtration was dried in vacuum to give the polymers containing gallium diiminate. (**GF**: 57 mg, 98%, **GC**: 160 mg, 89%, **GT**: 50 mg, 86%).

GF: M_n = 9,500, M_w/M_n = 1.9. ^{1}H NMR ($CDCl_3$): δ = 7.71–7.14 (24H, Ar-*H*), 5.55 (1H, br s, -C*H*=), 1.99 (2H), 1.25–1.03 (40H), 0.88–0.84 (6H), 0.62 (2H) ppm.

GC: M_n = 10,000, M_w/M_n = 1.8. ^{1}H NMR ($CDCl_3$): δ = 8.11–7.14 (24H, Ar-*H*), 5.58 (1H, br s, -C*H*=), 4.65 (1H), 2.10 (2H), 1.86 (2H), 1.25–1.04 (35H), 0.82–0.78 (10H) ppm.

GT: M_n = 8,800, M_w/M_n = 1.6. ^{1}H NMR ($CDCl_3$): δ = 7.45–7.11 (20H, Ar-*H*), 5.50 (1H, br s, -C*H*=), 2.49 (4H), 1.22 (40H), 0.89–0.86 (6H) ppm.

Scheme S1. Post-polymerization method for the synthesis of the polymers containing gallium diiminate

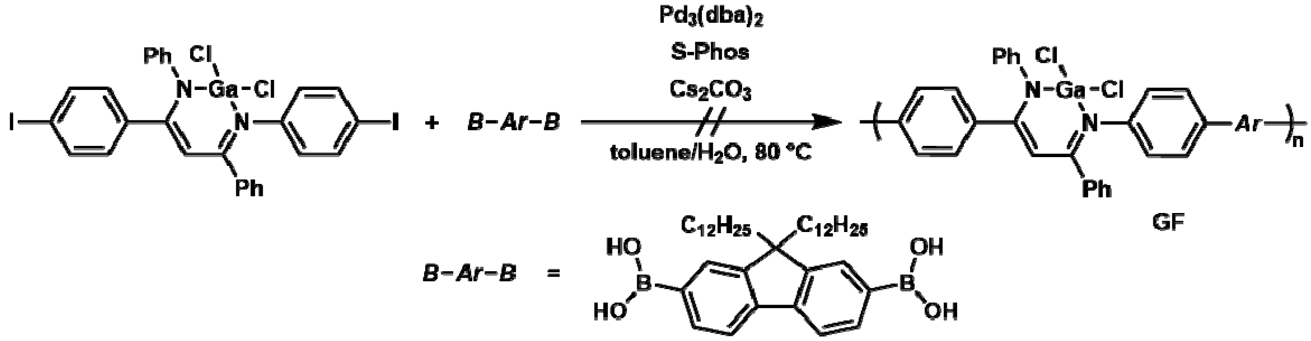

NMR Spectra

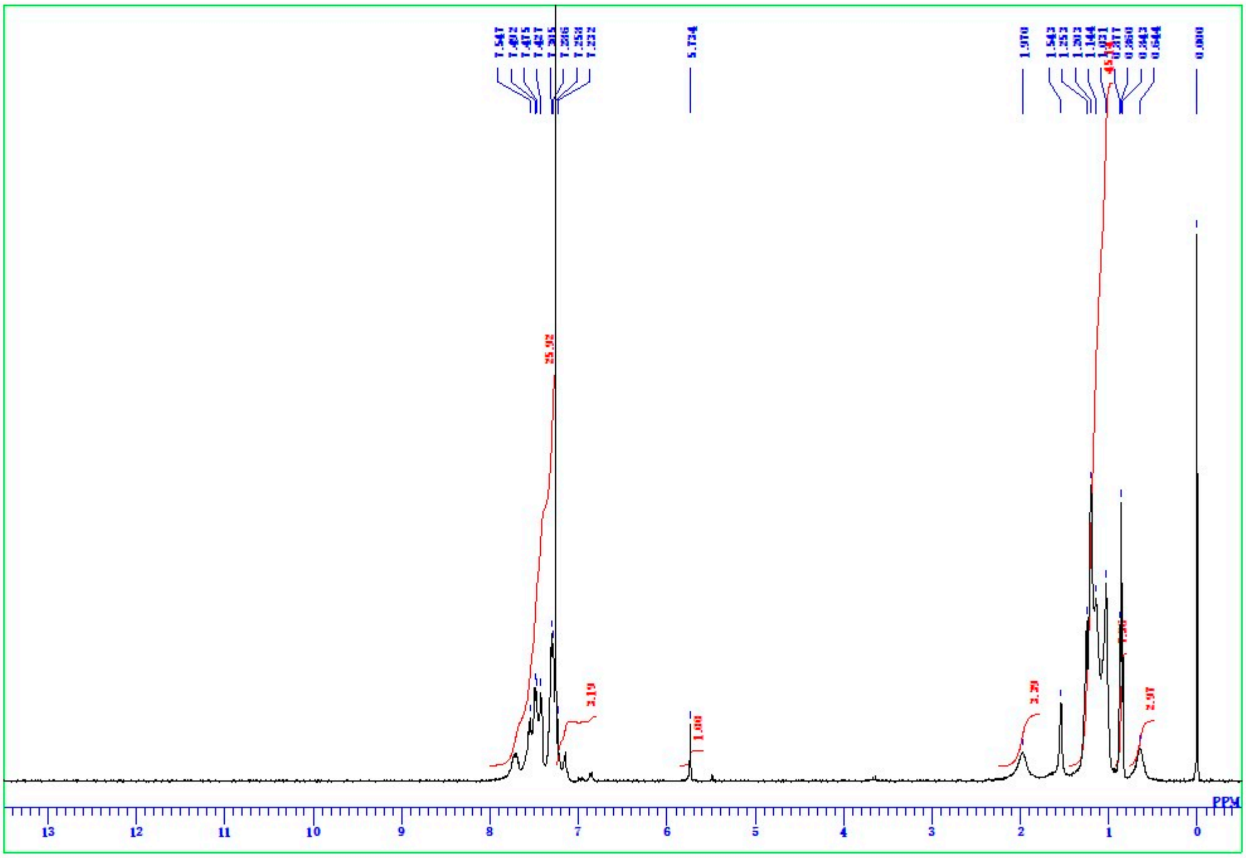

Figure S1. ^{1}H NMR spectrum of **BF** in $CDCl_3$.

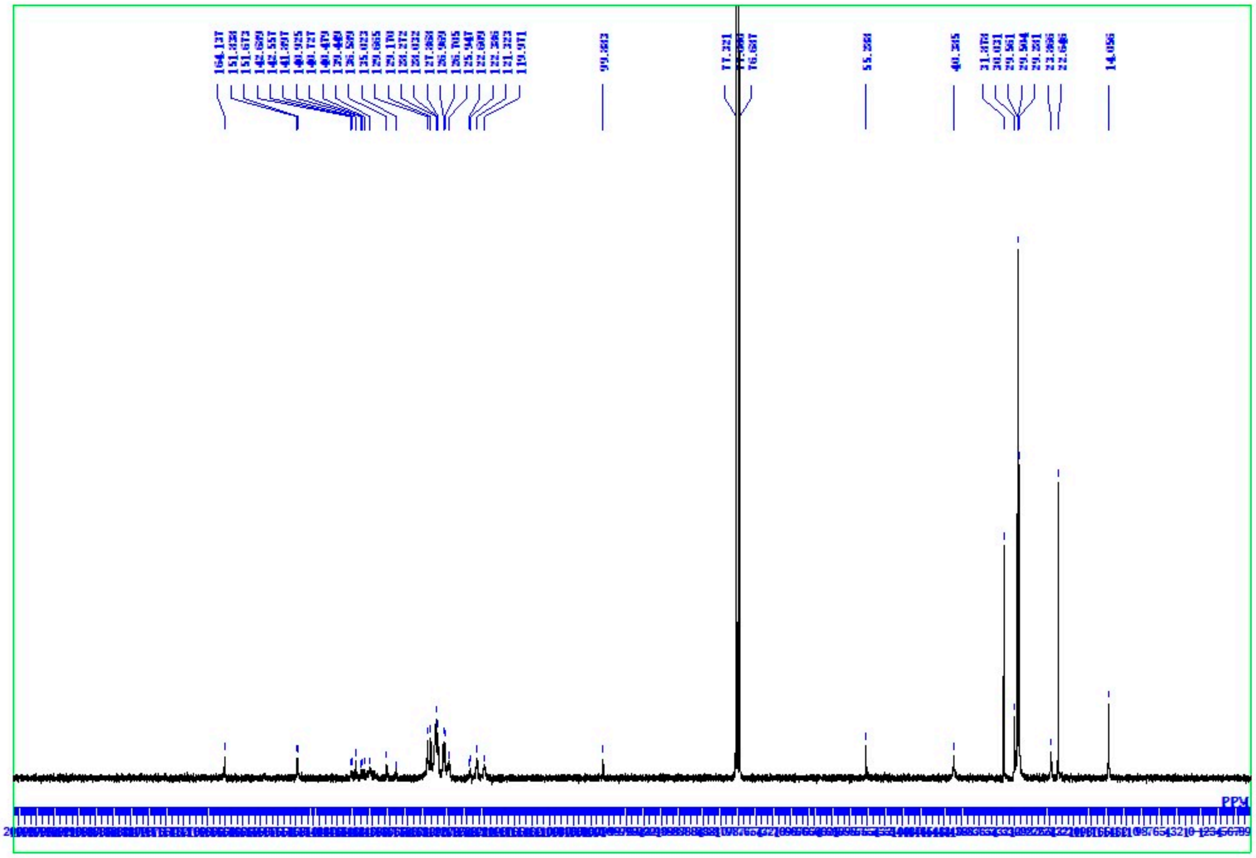

Figure S2. ^{13}C NMR spectrum of **BF** in $CDCl_3$.

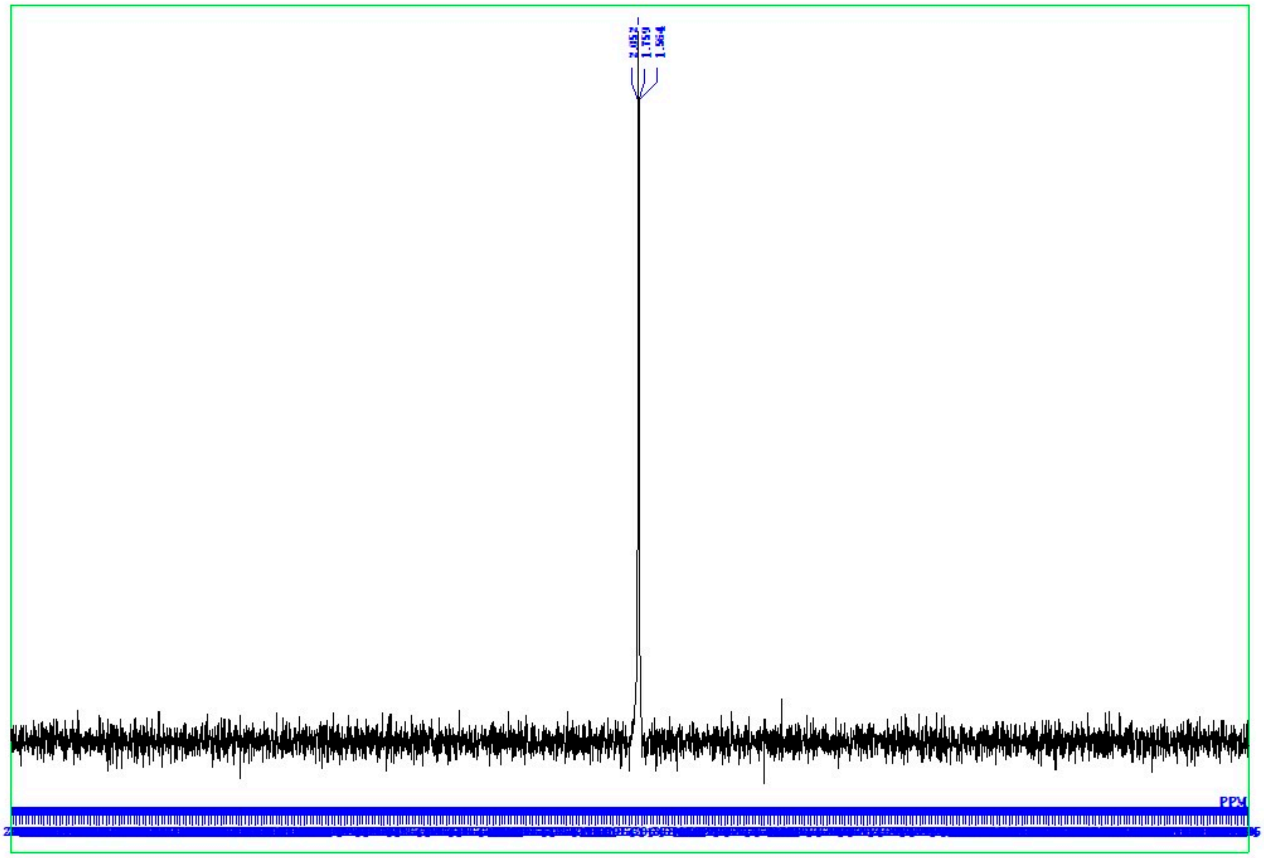

Figure S3. ^{11}B NMR spectrum of **BF** in $CDCl_3$.

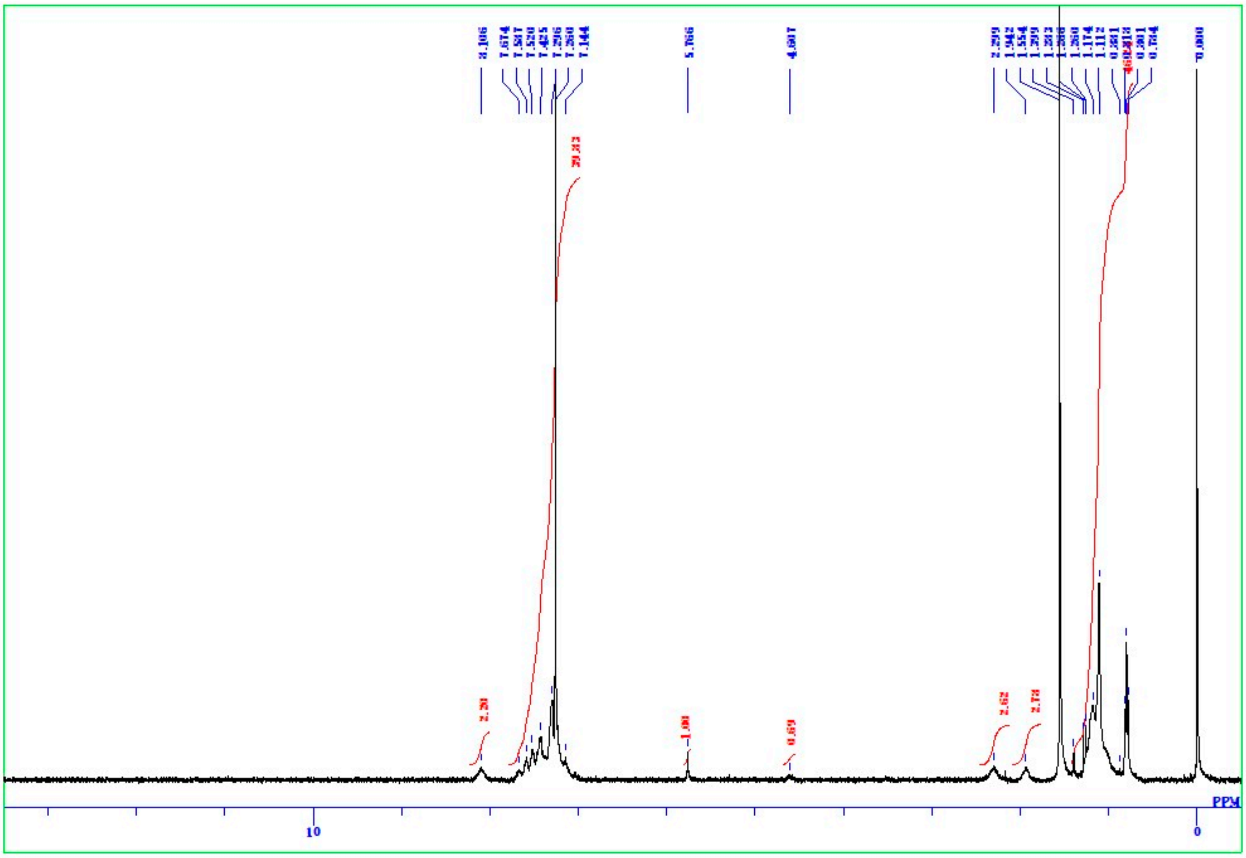

Figure S4. ^1H NMR spectrum of **BC** in $CDCl_3$.

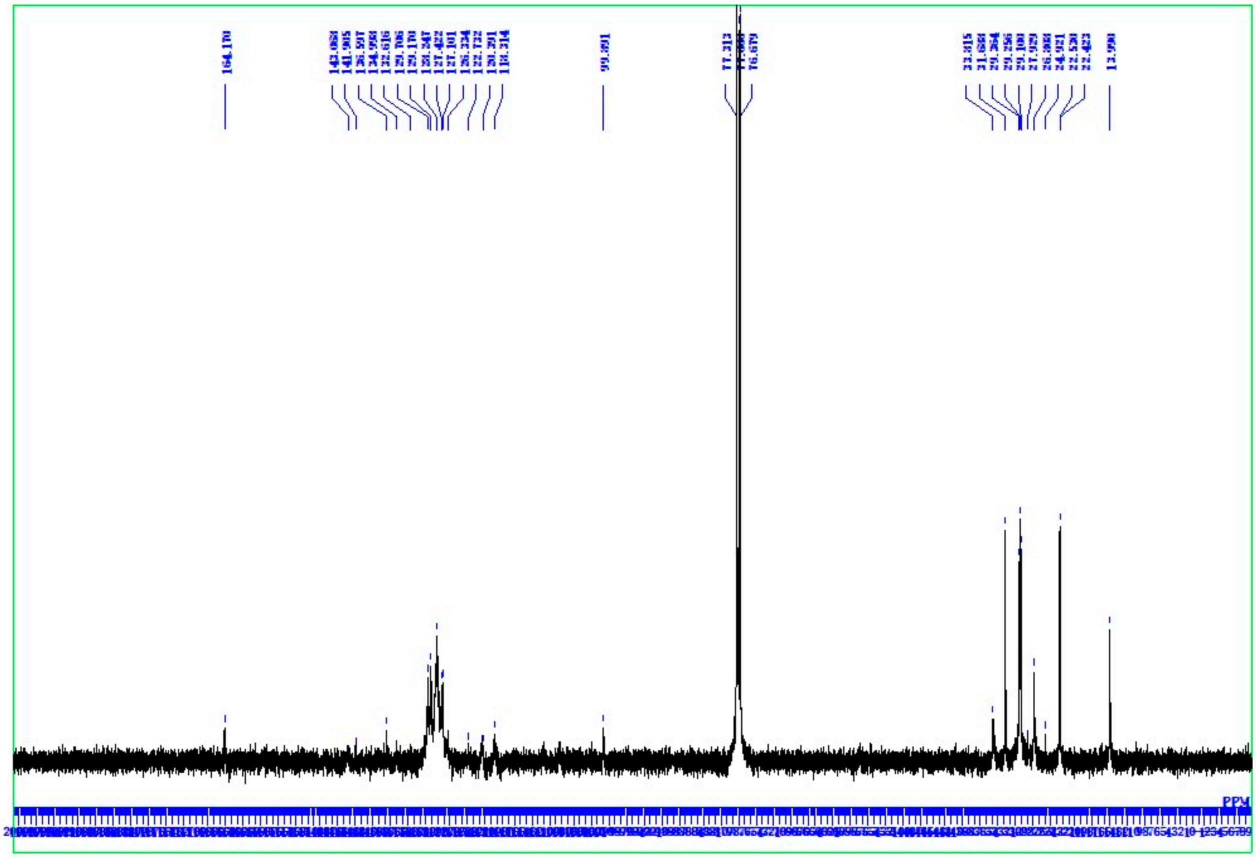

Figure S5. ^{13}C NMR spectrum of **BC** in $CDCl_3$.

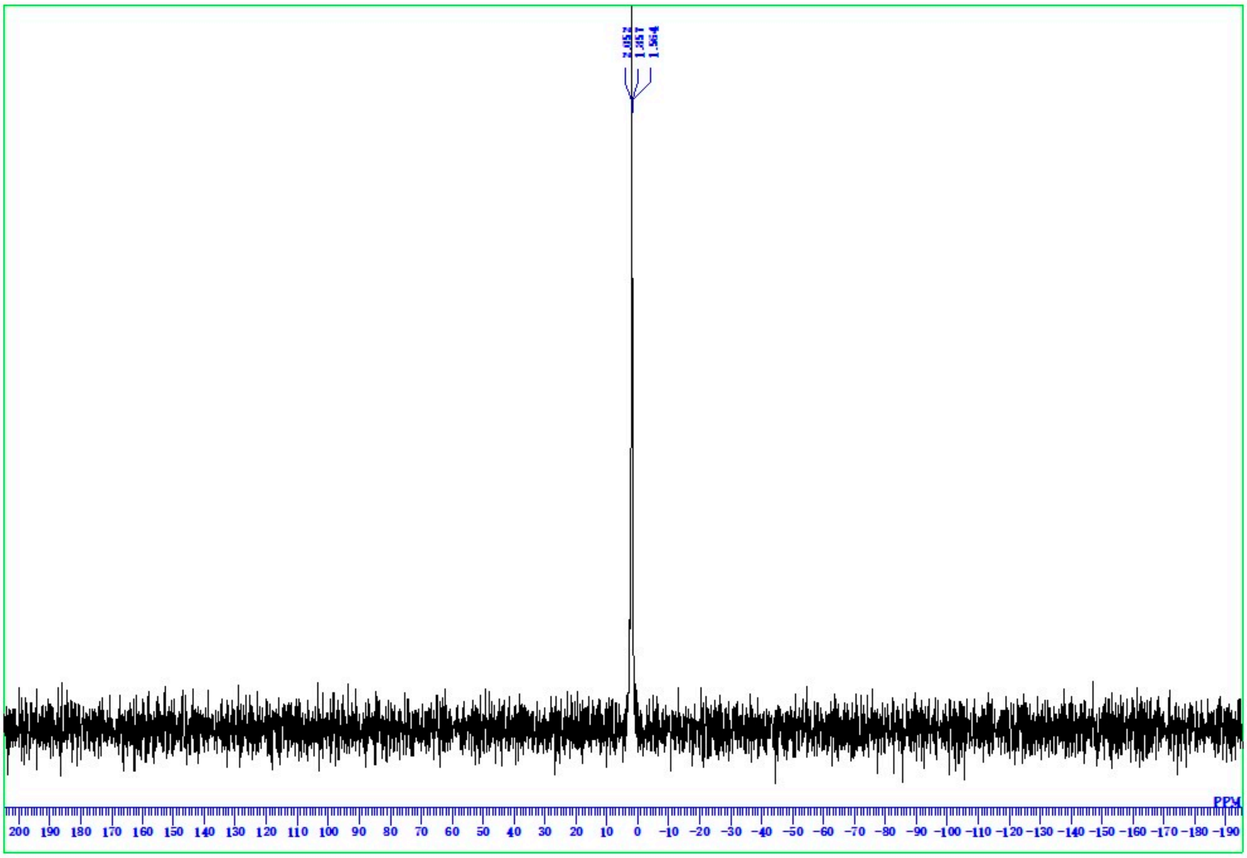

Figure S6. ^{11}B NMR spectrum of **BC** in $CDCl_3$.

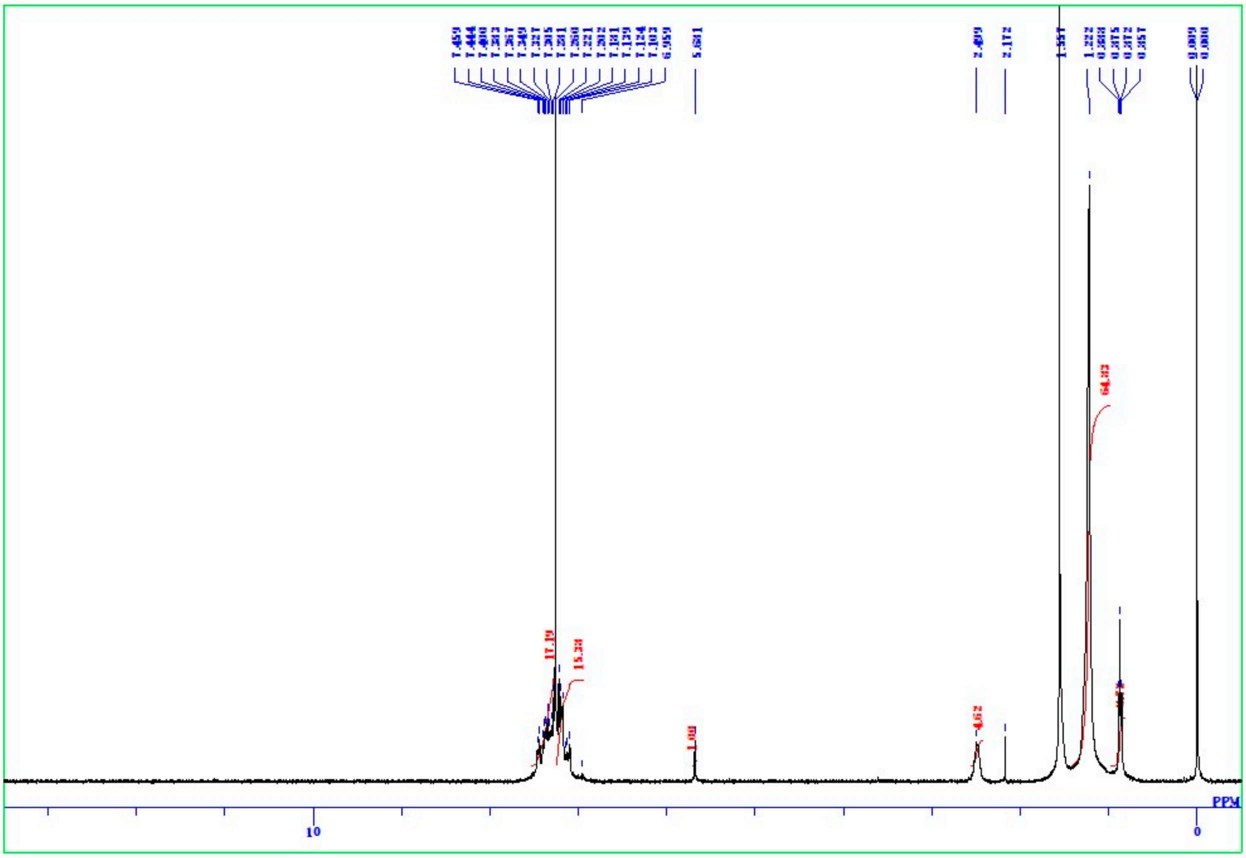

Figure S7. ^{1}H NMR spectrum of **BT** in $CDCl_3$.

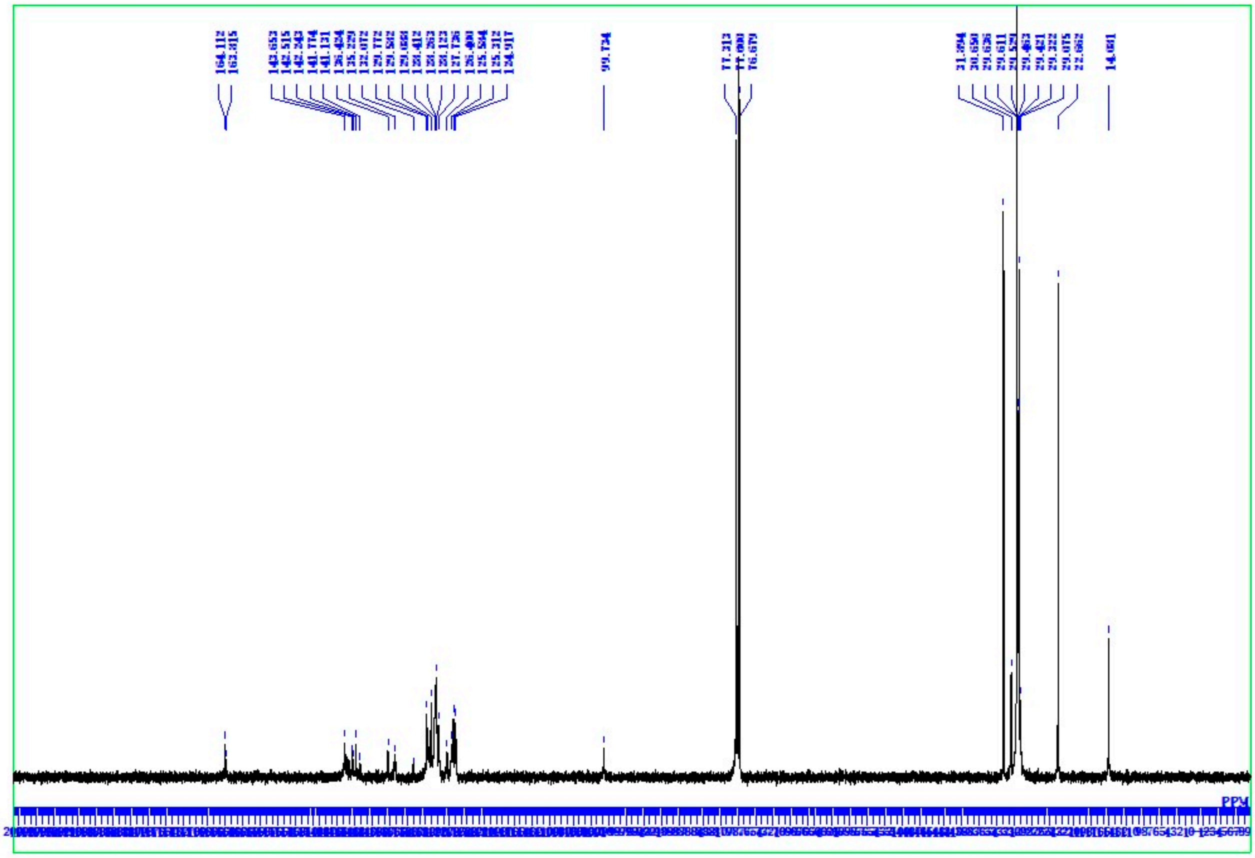

Figure S8. ^{13}C NMR spectrum of **BT** in $CDCl_3$.

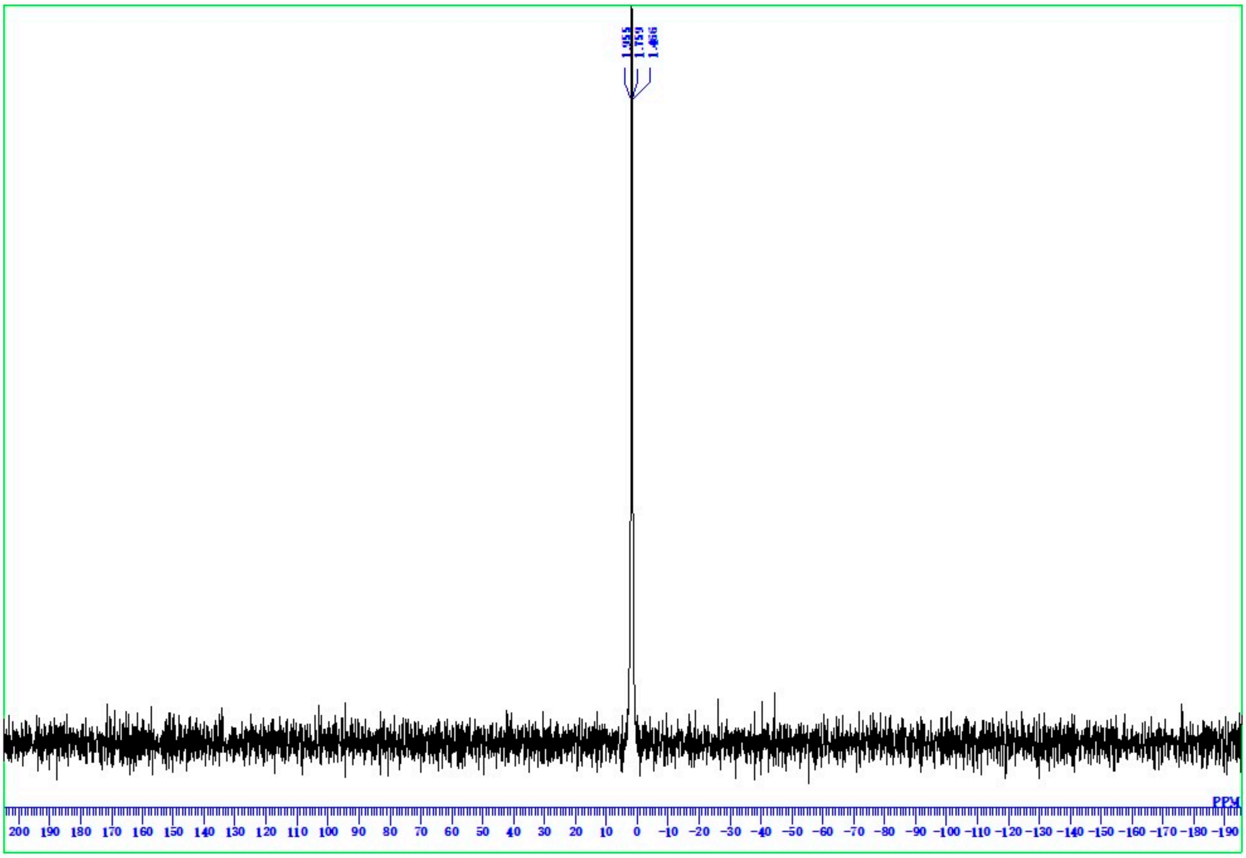

Figure S9. ^{11}B NMR spectrum of **BT** in $CDCl_3$.

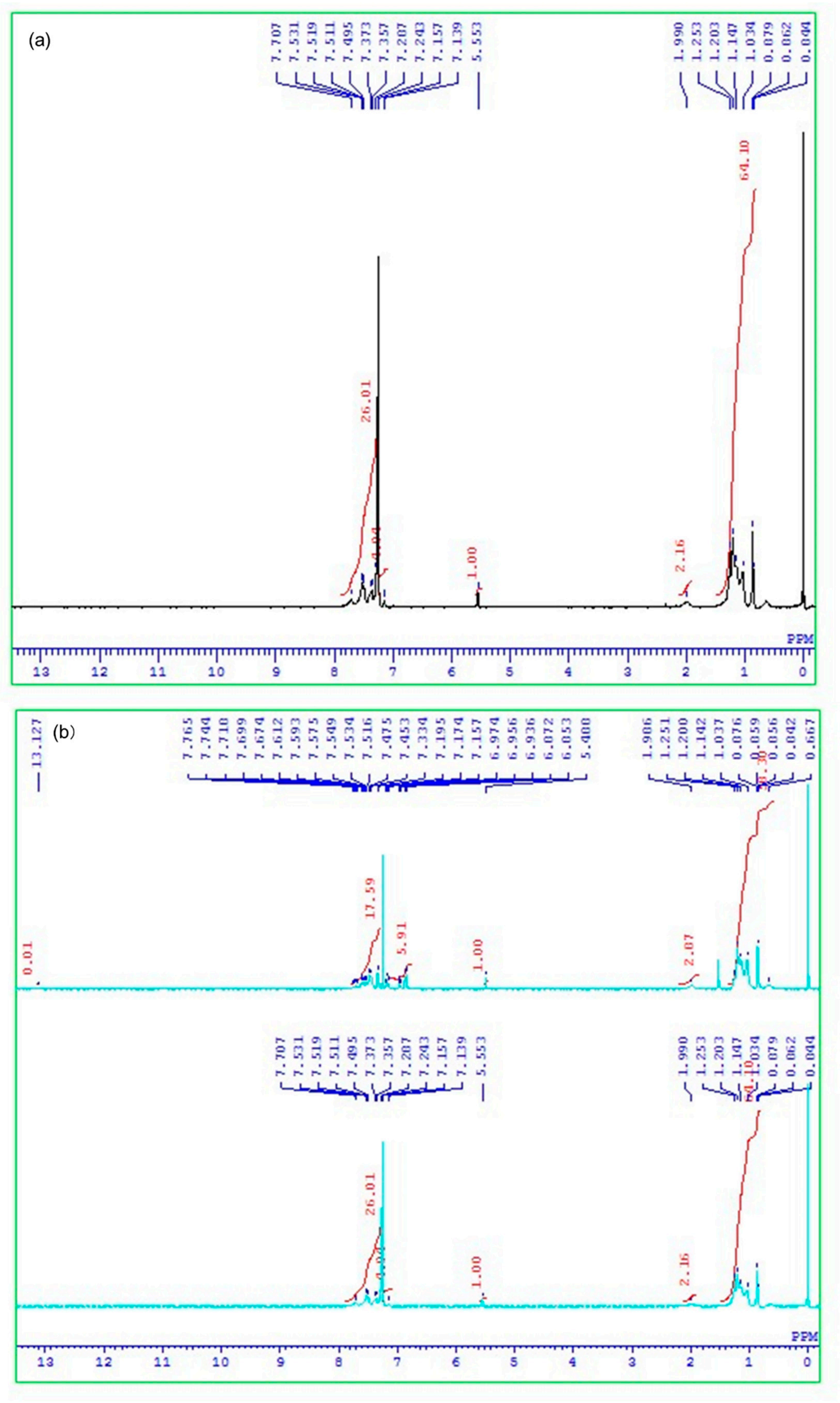

Figure S10. ^{1}H NMR spectra of (a) **GF** and comparison of the spectra between (b) **GF** (bottom) and **LF** (upper) in $CDCl_3$.

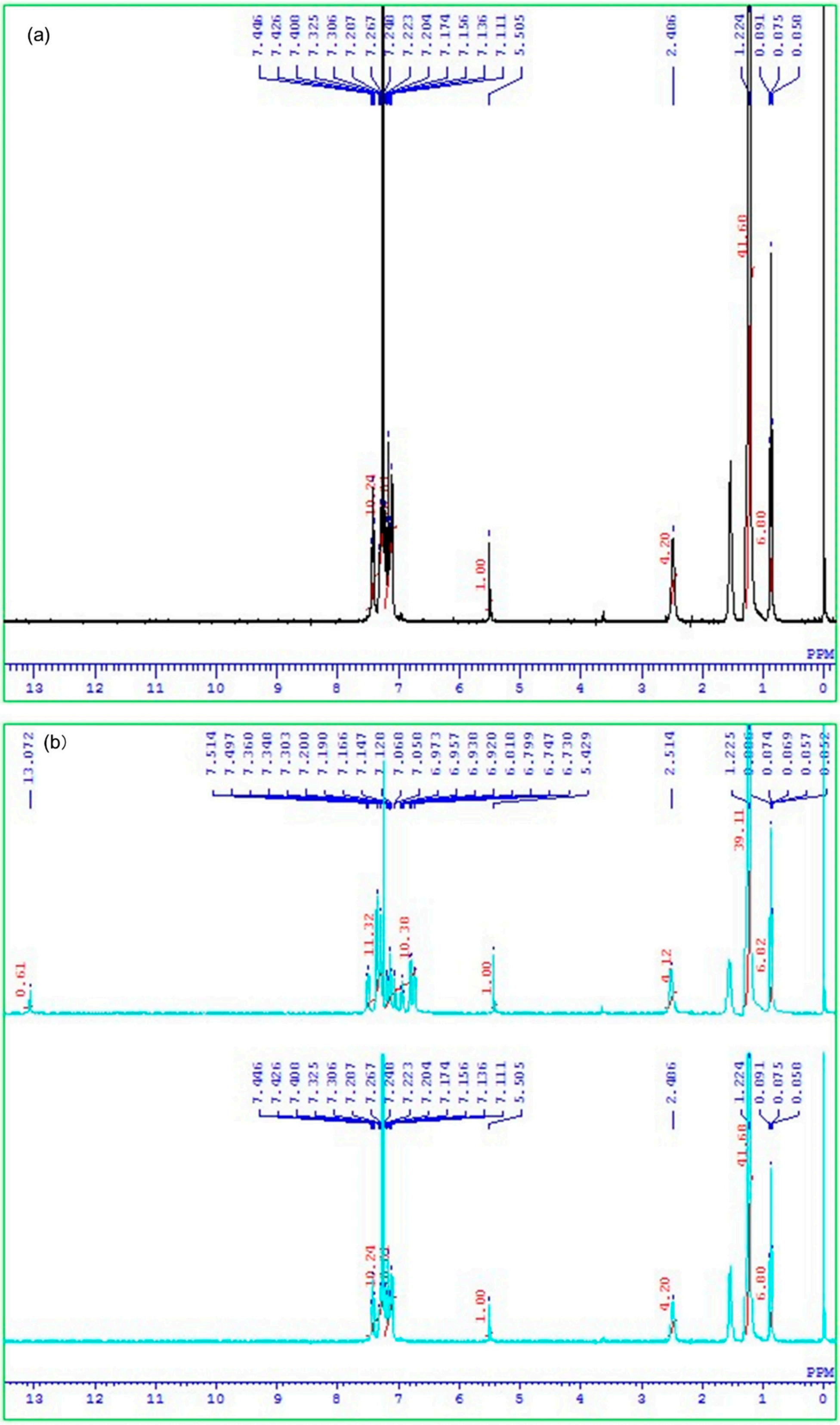

Figure S11. ^{1}H NMR spectra of (a) **GT** and comparison of the spectra between (b) **GT** (bottom) and **LT** (upper) in $CDCl_3$.

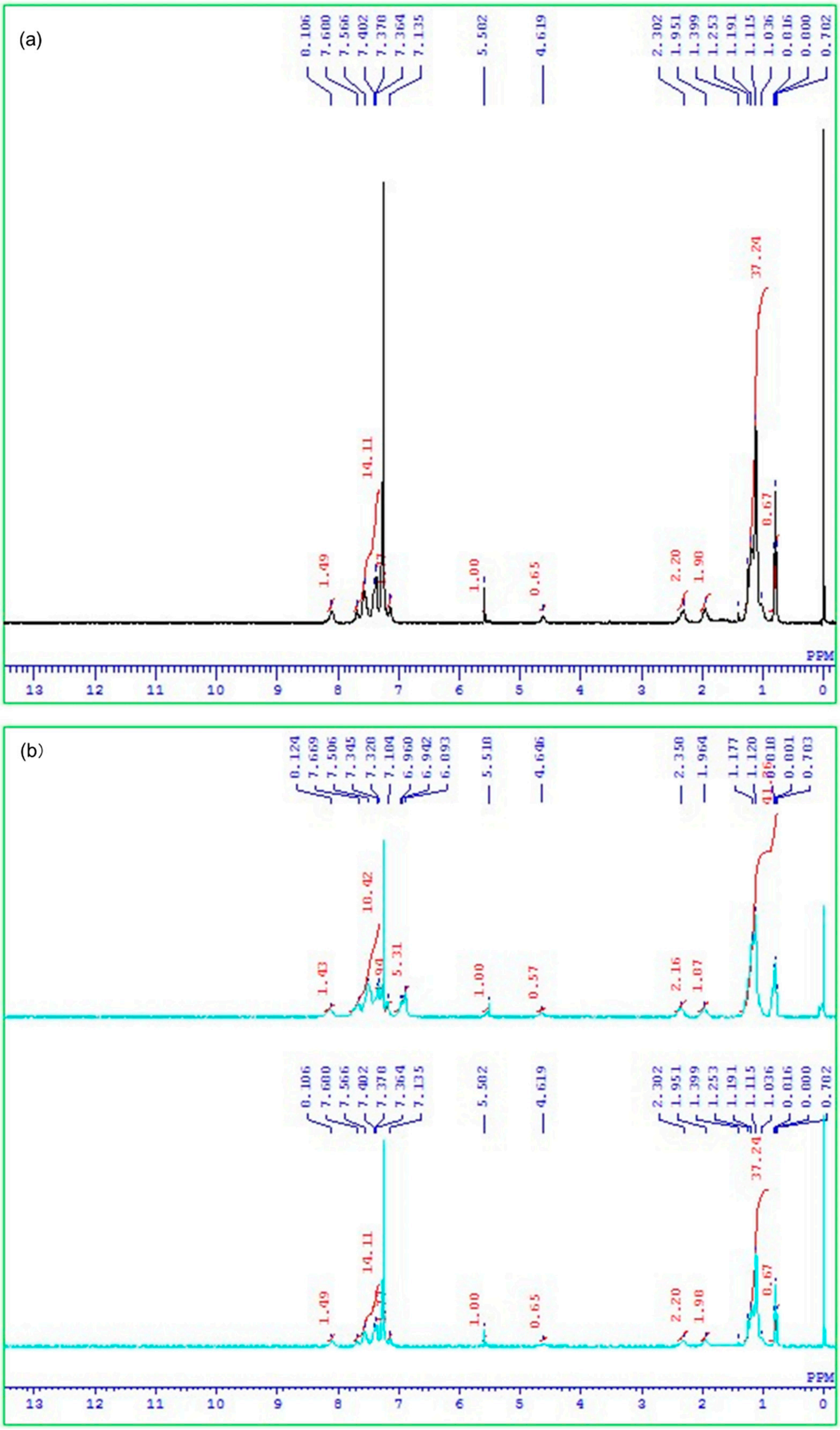

Figure S12. ^{1}H NMR spectra of (a) **GC** and comparison of the spectra between (b) **GC** (bottom) and **LC** (upper) in $CDCl_3$.

MALDI-TOF-MS Spectra

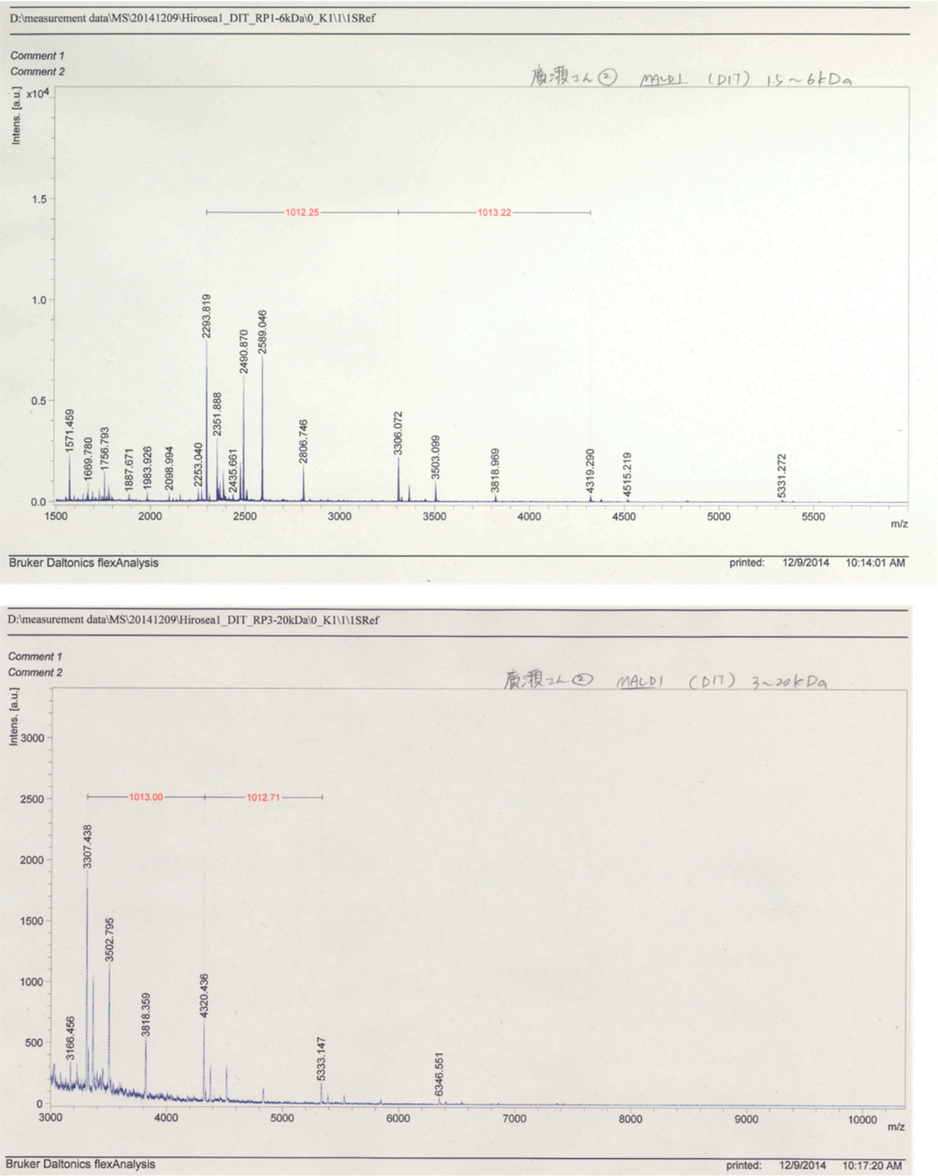

Figure S13. MALDI-TOF-MS spectra of **GF** using 1,8-dihydroxy-9,10-dihydroanthracene-9-one (DIT) as a matrix.

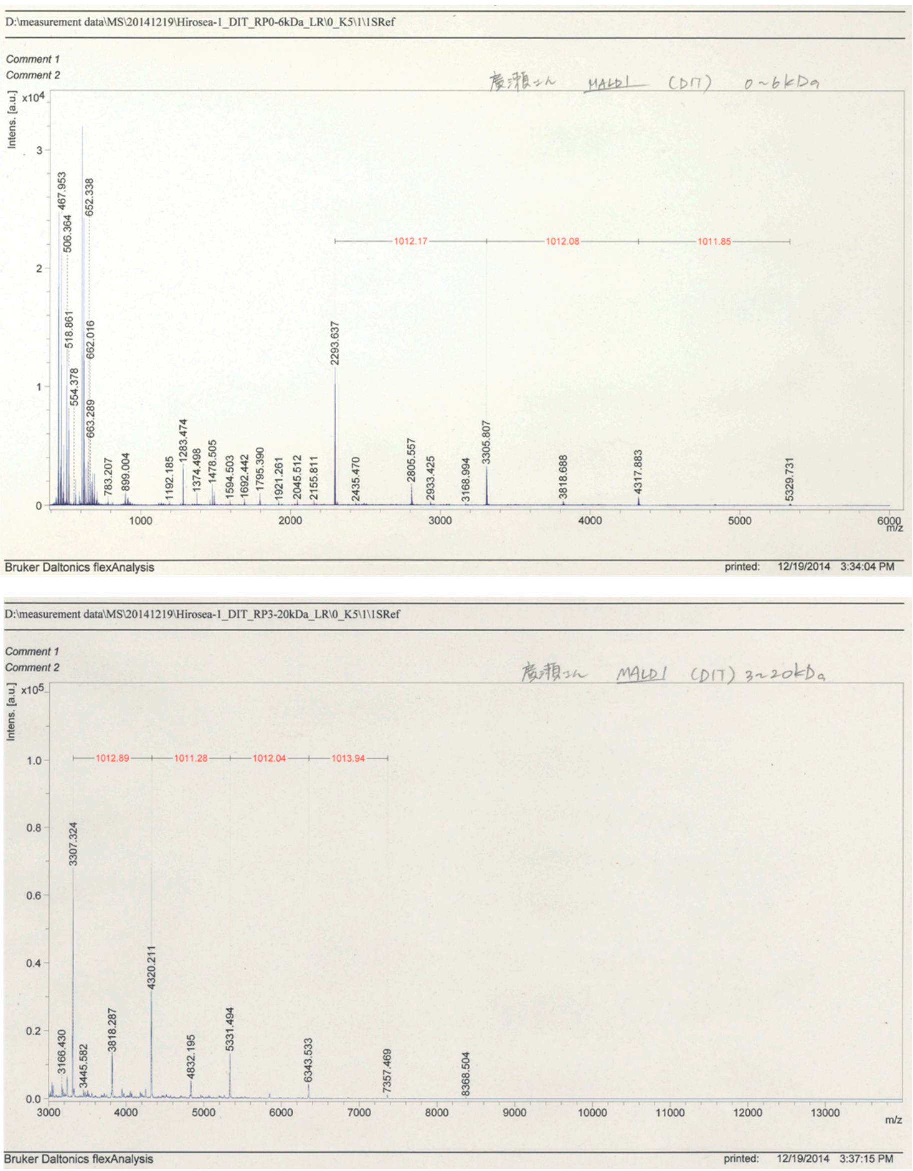

Figure S14. MALDI-TOF-Mass spectra of **GT** using DIT as a matrix.

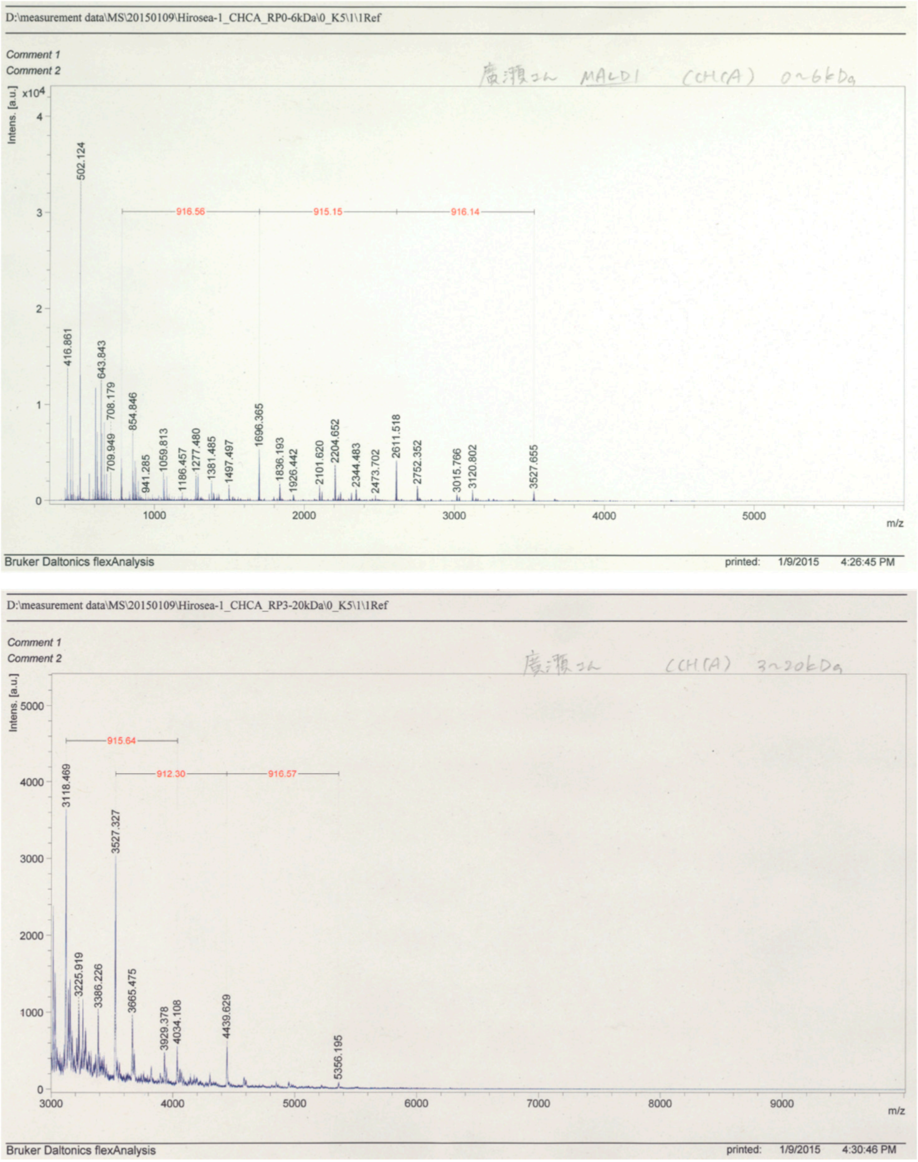

Figure S15. MALDI-TOF-Mass spectra of **GC** using α-cyano-4-hydroxycinnamic acid (CHCA) as a matrix.

FT-IR Spectra

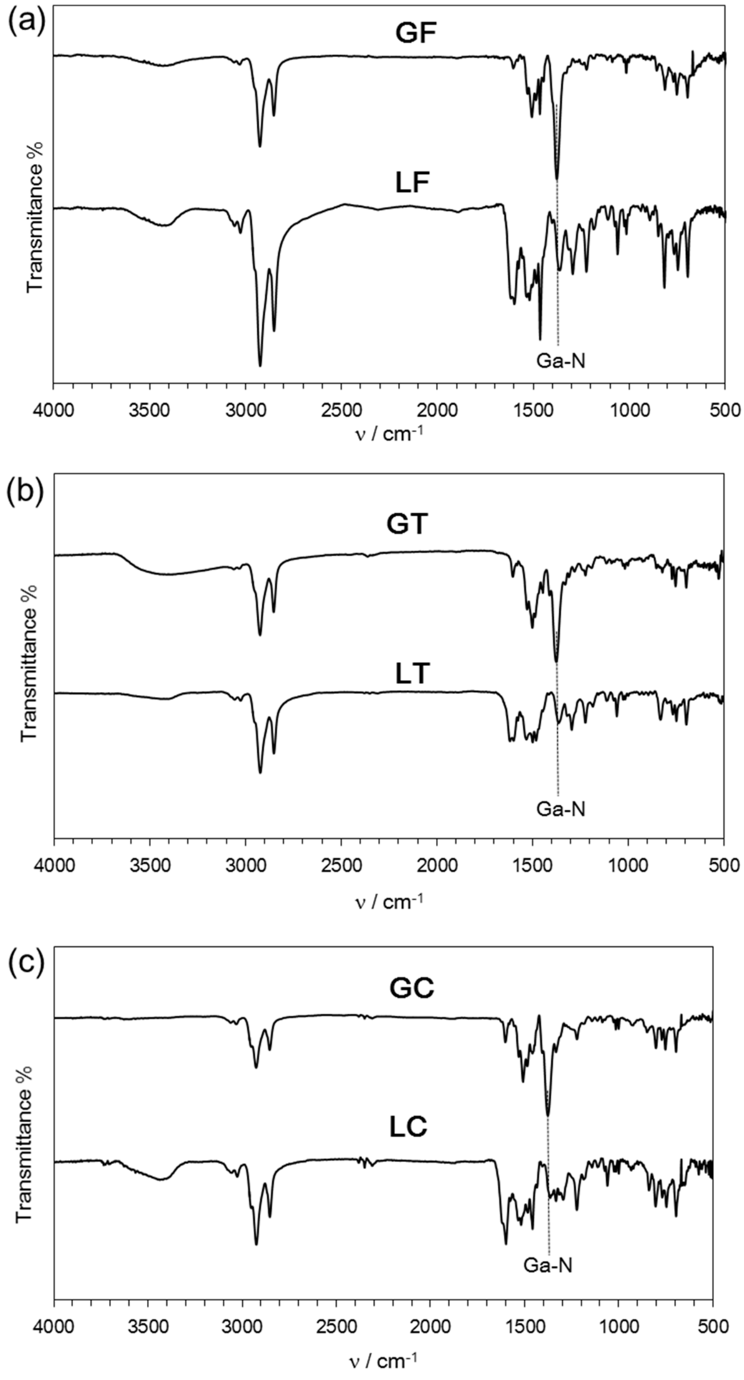

Figure S16. FT-IR spectra of the polymers (film on KBr).

XRF Profiles

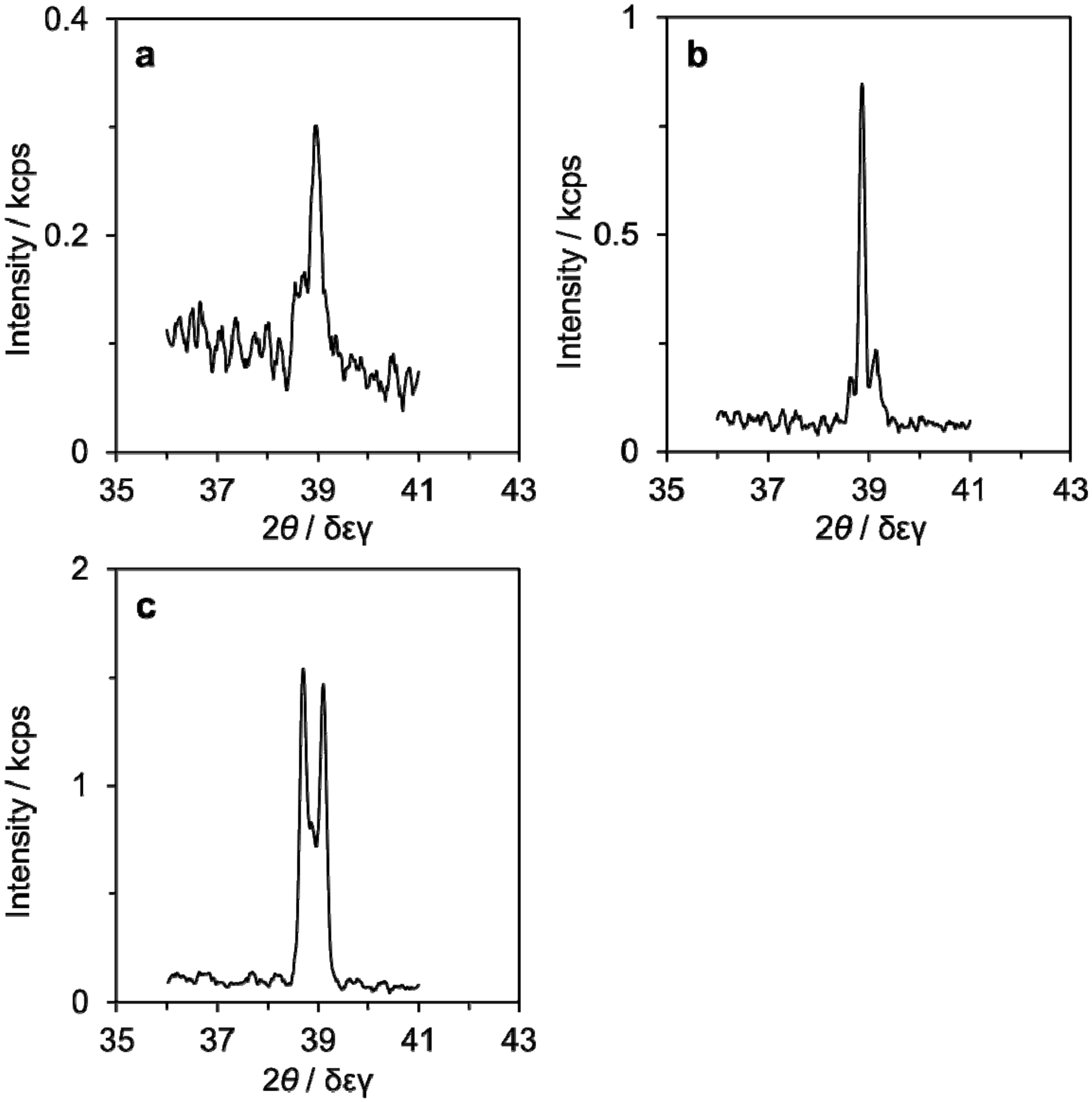

Figure S17. The results of XRF analyses. (a: **GF**, b: **GT**, c: **GC**)

TGA Profiles

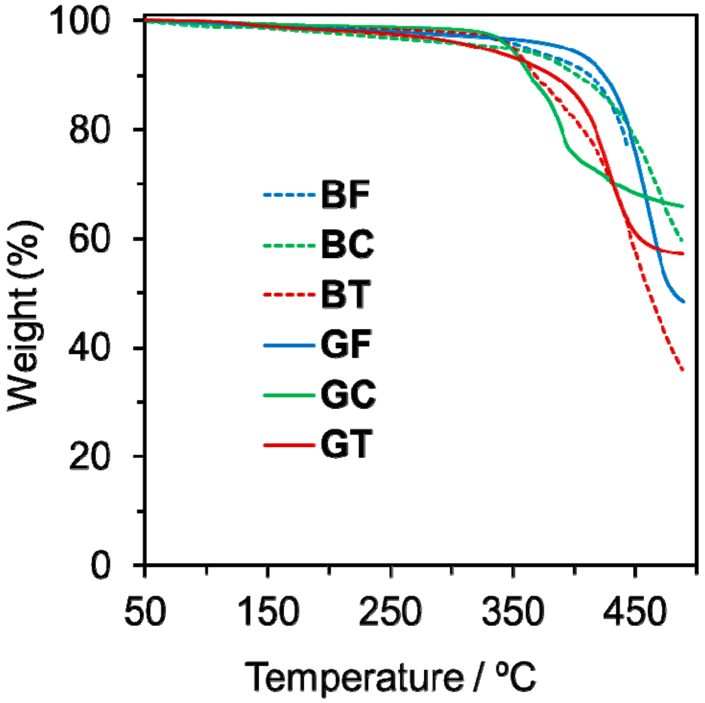

Figure S18. TGA profiles of the synthesized polymers.

DSC Profiles

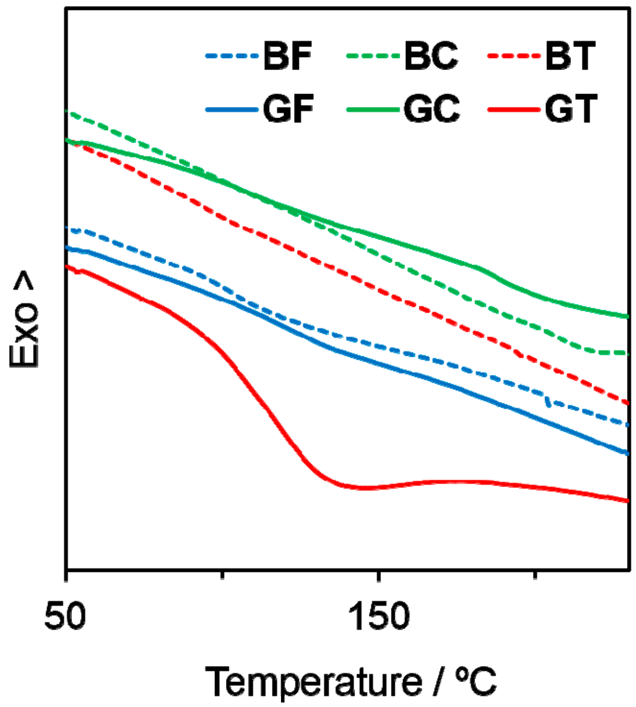

Figure S19. DSC profiles of the synthesized polymers.

Cyclic Voltammograms

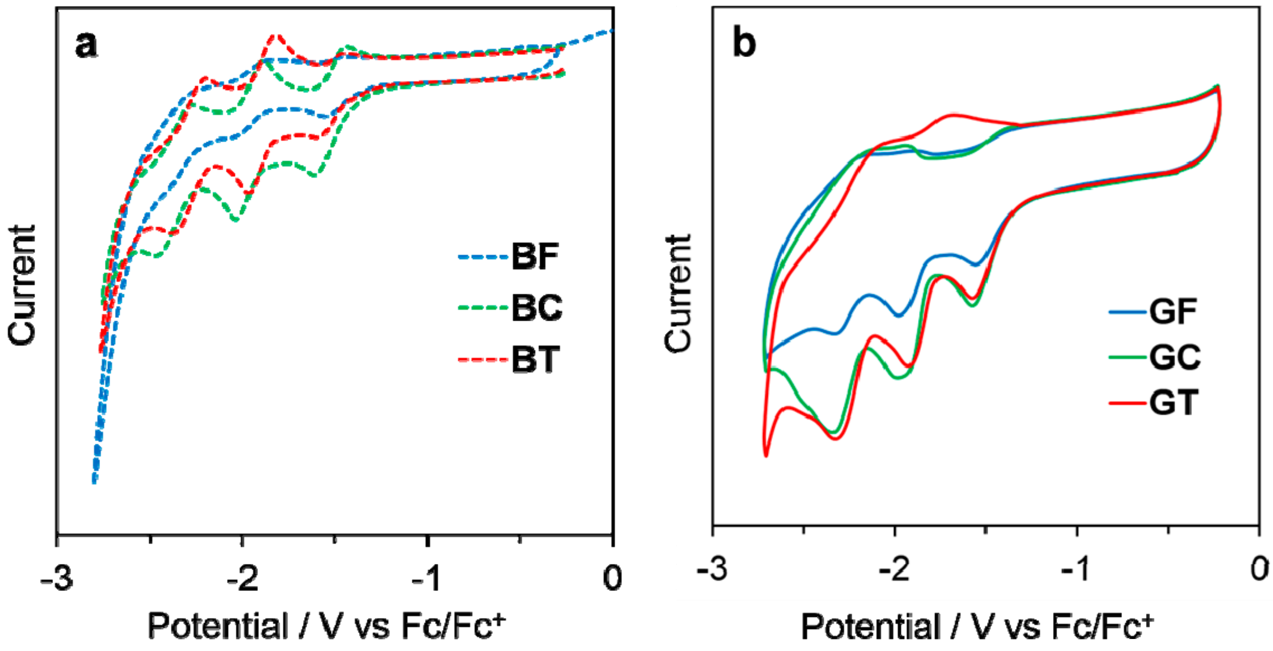

Figure S20. Cyclic voltammograms of the synthesized polymers; (a) **BF**, **BC** and **BT** and (b) **GF**, **GC** and **GT**.

UV–vis Absorption Spectra of the Polymers in the Film States

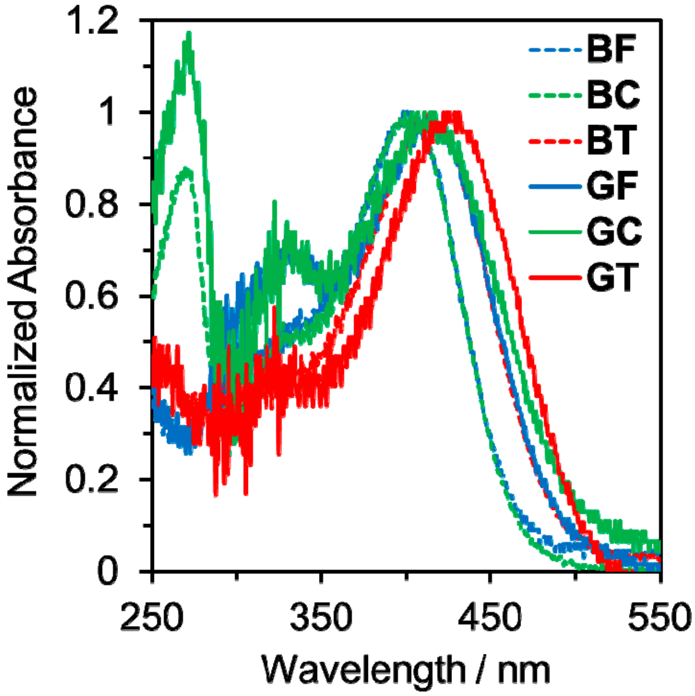

Figure S21. Normalized UV–vis absorption spectra of the spin-coated thin films of the synthesized polymers.

Lippert–Mataga Plots

Lippert–Mataga plots were conducted with the following formula.[3]

$$\tilde{\nu}_{\mathrm{abs}} - \tilde{\nu}_{\mathrm{PL}} = \frac{2\Delta f}{4\pi\varepsilon_0 hca^3}(\mu_{\mathrm{E}} - \mu_{\mathrm{G}})^2 + \mathrm{const.}$$

where ε_0 is the permittivity of vacuum h equals Planck's constant, c equals the velocity of light in a vacuum, a is the Onsager cavity radius, n and ε are the refractive index and the dielectric constant of the solvent, $\tilde{\nu}_{\mathrm{abs}}$ and $\tilde{\nu}_{\mathrm{PL}}$ are the absorption and photoluminescence wavenumber, μ_{E} and μ_{G} are the dipole moments in the excited and the ground state, and Δf is the orientation polarizability defined as below, respectively.

$$\Delta f = \frac{\varepsilon - 1}{2\varepsilon + 1} - \frac{n^2 - 1}{2n^2 + 1}$$

Hence the slope derived from a plot of Stokes-shifts ($\tilde{\nu}_{\mathrm{abs}} - \tilde{\nu}_{\mathrm{PL}}$) as a function of Δf can be expressed as

$$\mathrm{slope} = \frac{2}{4\pi\varepsilon_0 hca^3}(\mu_{\mathrm{E}} - \mu_{\mathrm{G}})^2$$

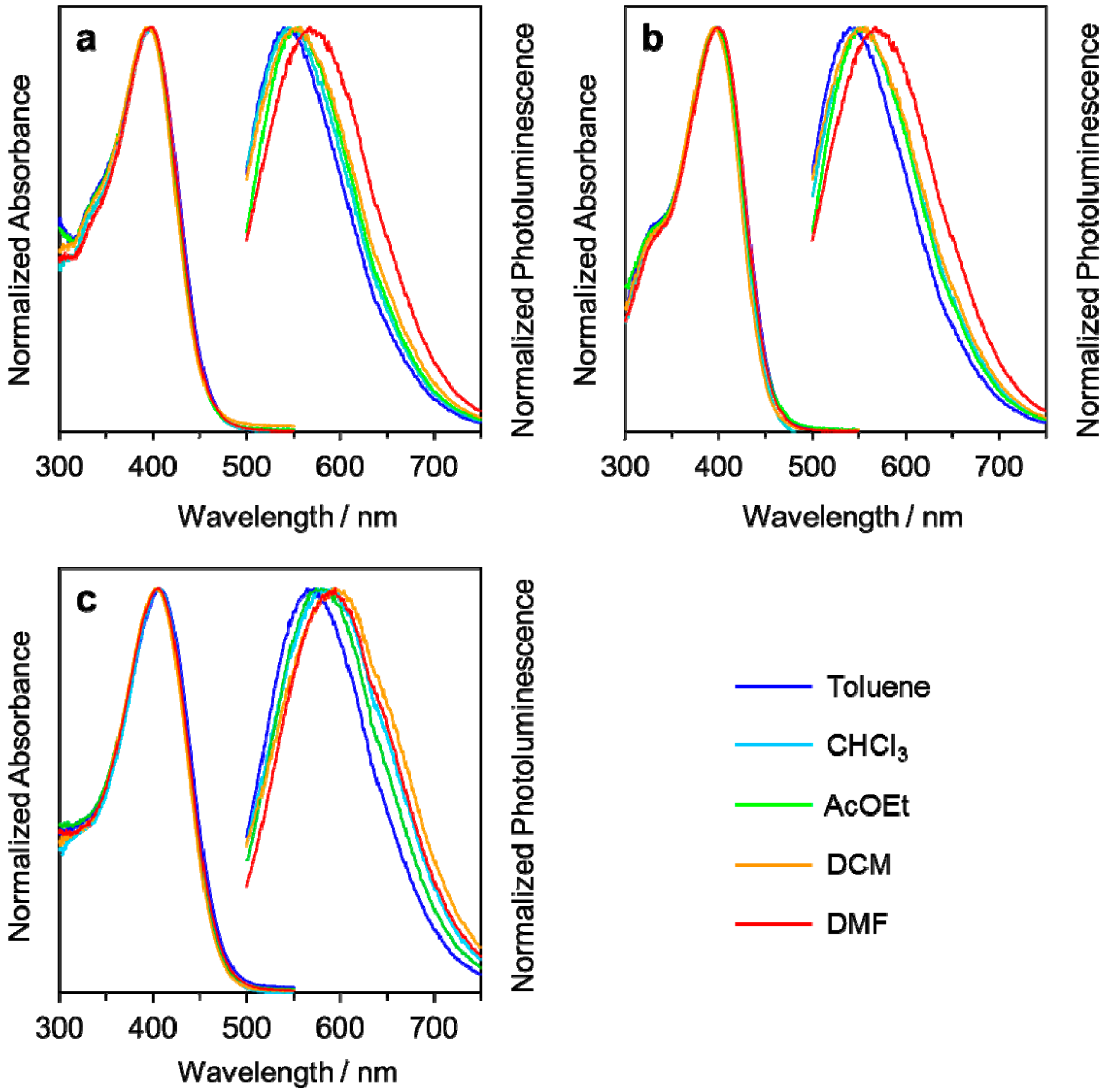

Figure S22. Normalized UV–vis absorption and photoluminescence spectra of **BF**, **BC** and **BT** in various solvents (1.0 × 10^{-5} M).

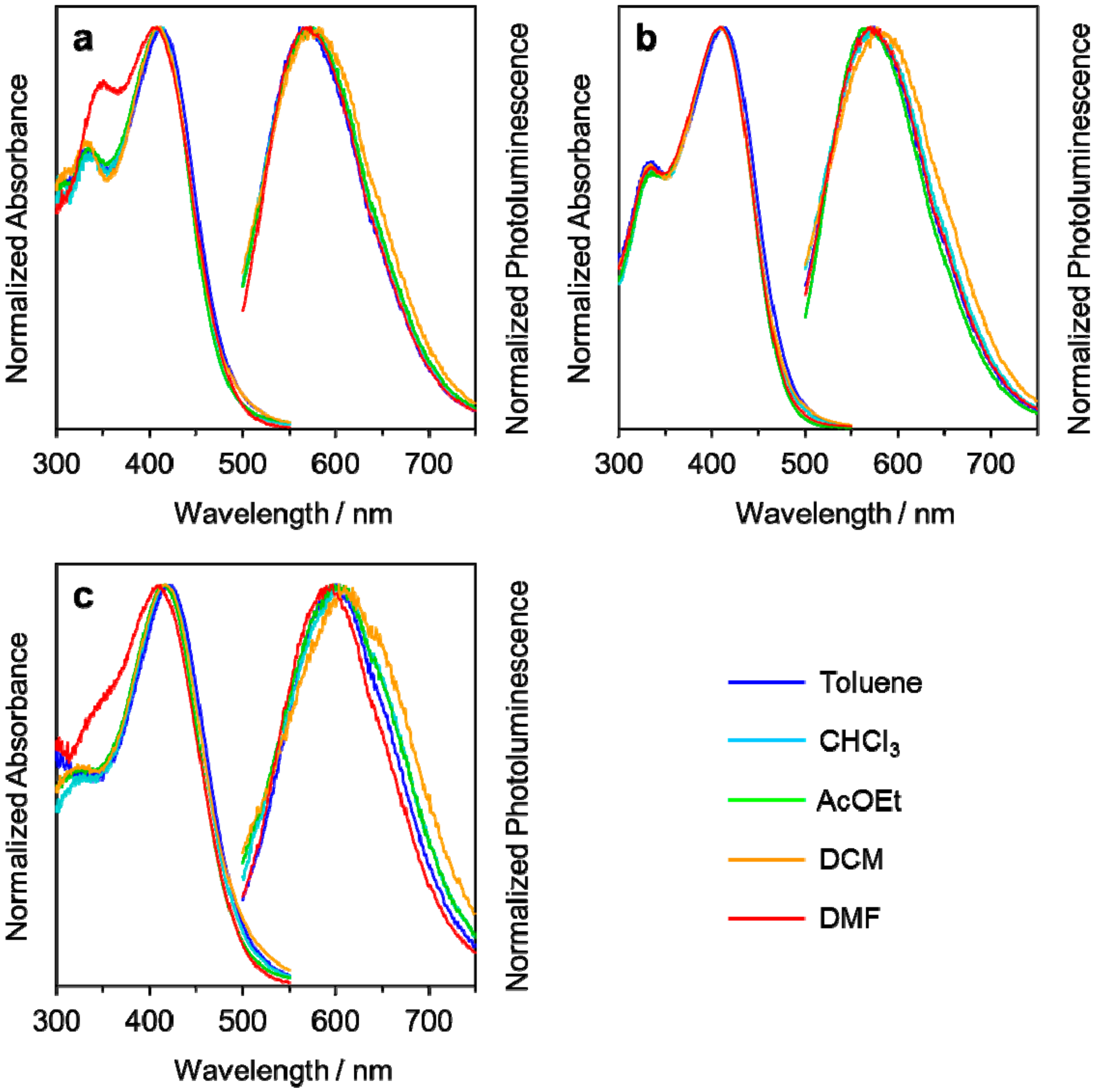

Figure S23. Normalized UV–vis absorption and photoluminescence spectra of **GF**, **GC** and **GT** in various solvents (1.0×10^{-5} M).

Table S1. Photophysical properties of **BF** in different solvents[a]

Solvent	λ_{abs} / nm	λ_{PL} / nm	Stokes-shift / cm^{-1}
Toluene	398	540	6640
$CHCl_3$	396	545	6940
AcOEt	396	555	7270
CH_2Cl_2	393	557	7490
DMF	397	568	7580

[a] Measured in 1.0×10^{-5} M solutions of each solvent.

Table S2. Photophysical properties of **BC** in different solvents[a]

Solvent	λ_{abs} / nm	λ_{PL} / nm	Stokes-shift / cm^{-1}
Toluene	398	540	6640
$CHCl_3$	397	545	6870
AcOEt	397	555	7170
CH_2Cl_2	397	557	7240
DMF	400	568	7430

[a] Measured in 1.0×10^{-5} M solutions of each solvent.

Table S3. Photophysical properties of **BT** in different solvents[a]

Solvent	λ_{abs} / nm	λ_{PL} / nm	Stokes-shift / cm^{-1}
Toluene	407	573	7120
$CHCl_3$	404	581	7540
AcOEt	405	594	7860
CH_2Cl_2	405	600	8020
DMF	407	573	7120

[a] Measured in 1.0×10^{-5} M solutions of each solvent.

Table S4. Photophysical properties of **GaF** in different solvents[a]

Solvent	λ_{abs} / nm	λ_{PL} / nm	Stokes-shift / cm^{-1}
Toluene	412	565	6570
$CHCl_3$	407	576	7240
AcOEt	408	573	7060
CH_2Cl_2	411	581	7120
DMF	408	574	7090

[a] Measured in 1.0×10^{-5} M solutions of each solvent.

Table S5. Photophysical properties of **GaC** in different solvents[a]

Solvent	λ_{abs} / nm	λ_{PL} / nm	Stokes-shift / cm^{-1}
Toluene	411	575	6940
$CHCl_3$	408	572	7030
AcOEt	409	571	6970
CH_2Cl_2	411	578	7030
DMF	408	570	6970

[a] Measured in 1.0×10^{-5} M solutions of each solvent.

Table S6. Photophysical properties of **GaT** in different solvents[a]

Solvent	λ_{abs} / nm	λ_{PL} / nm	Stokes-shift / cm^{-1}
Toluene	423	601	7000
$CHCl_3$	418	605	7390
AcOEt	417	598	7290
CH_2Cl_2	418	604	7400
DMF	412	597	7520

[a] Measured in 1.0×10^{-5} M solutions of each solvent.

Table S7. Results of Lippert–Mataga plots and estimated electric dipole moments

Compound	Slope / cm^{-1}	a / Å[a]	μ_G / Debye[b]	$\mu_G - \mu_E$ / Debye	μ_E / Debye
**BF**	3830	7.46	4.90	12.6	17.5
**BC**	3070	7.30	6.11	10.9	17.0
**BT**	3533	6.97	4.41	10.9	15.3
**GaF**	1130	7.70	6.72	7.16	13.9
**GaC**	148	7.74	8.19	2.61	10.8
**GaT**	1840	7.45	7.02	8.69	15.7

[a] Effective radii of the Onsager cavity were estimated from the molecular dimensions of each model compound obtained from the DFT calculations at B3LYP/6-31G(d,p) level of theory and from the assumption of a spherical shape of this cavity with Gaussian09 keyword "volume". [b] Electric dipole moments in a ground state were estimated from the optimized structure of each model compound obtained from the DFT calculations at B3LYP/6-31G(d,p) level of theory.

DFT Calculations

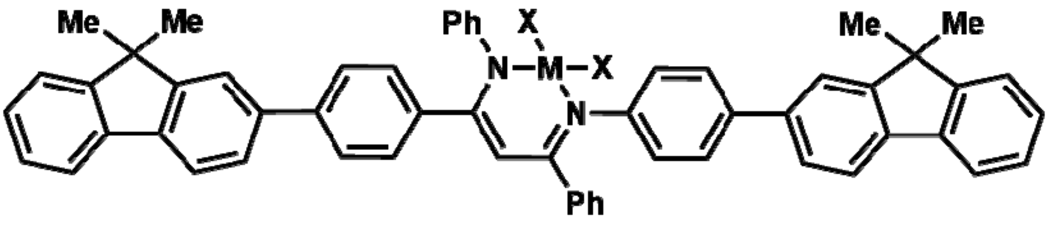

MBF, MGF

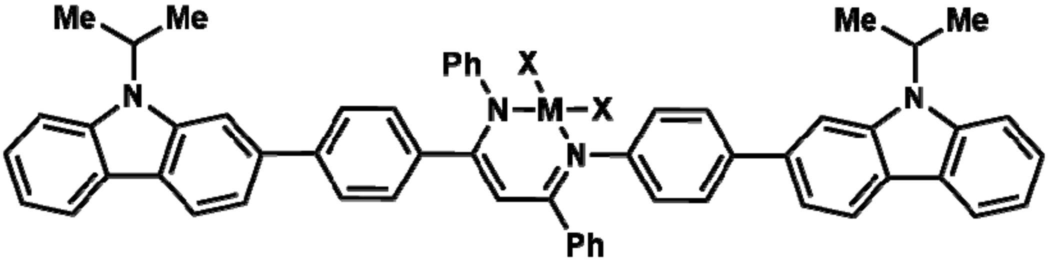

MBC, MGC

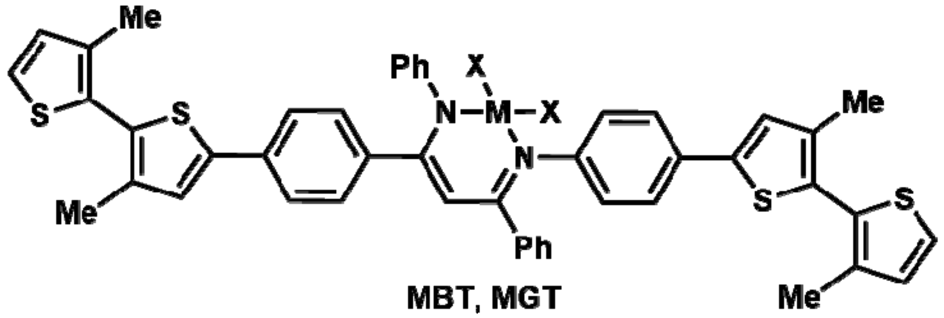

MBT, MGT

Figure S24. Chemical structures of the model compounds for the DFT calculations.

Table S8. Calculated frontier orbital energies of the model compounds for the synthesized polymers [a]

	HOMO / eV	LUMO / eV
**MBF**	–5.35	–1.89
**MBC**	–5.26	–1.85
**MBT**	–5.28	–1.98
**MGF**	–5.46	–2.17
**MGC**	–5.33	–2.09
**MGT**	–5.42	–2.22

[a] Calculated with a density functional theory at a B3LYP level of theory with a 6-31G(d,p) basis set.

Table S9. Calculated compositions of $S_0 \rightarrow S_1$ electronic transitions for the model compounds[a]

	f^b<br>Compositions	Energy / eV,<br>Wavelength / nm
**MBF**	0.6948<br>HOMO → LUMO (0.69342)	3.11, 399
**MBC**[c]	0.7203<br>HOMO–1 → LUMO (0.10671)<br>HOMO → LUMO (0.68709)	3.07, 404
**MBT**	0.6041<br>HOMO → LUMO (0.69719)	2.94, 421
**MGF**	0.5495<br>HOMO → LUMO (0.69463)	2.93, 423
**MGC**[c]	0.5019<br>HOMO–1 → LUMO (0.68017)<br>HOMO → LUMO (–0.15399)	2.93, 423
**MGT**	0.4342<br>HOMO → LUMO (0.69762)	2.81, 438

[a] Calculated with a time-dependent density functional theory at a B3LYP level of theory with a 6-31G(d,p) basis set. [b] An oscillator strength of a transition. [c] The orbital distributions of HOMO and HOMO–1 of **MBC** and **MGC** were mainly originated from those of each carbazole unit. The energy gaps between them were respectively estimated to be 0.024 eV and 0.055 eV, where these two molecular orbitals are almost degenerated.

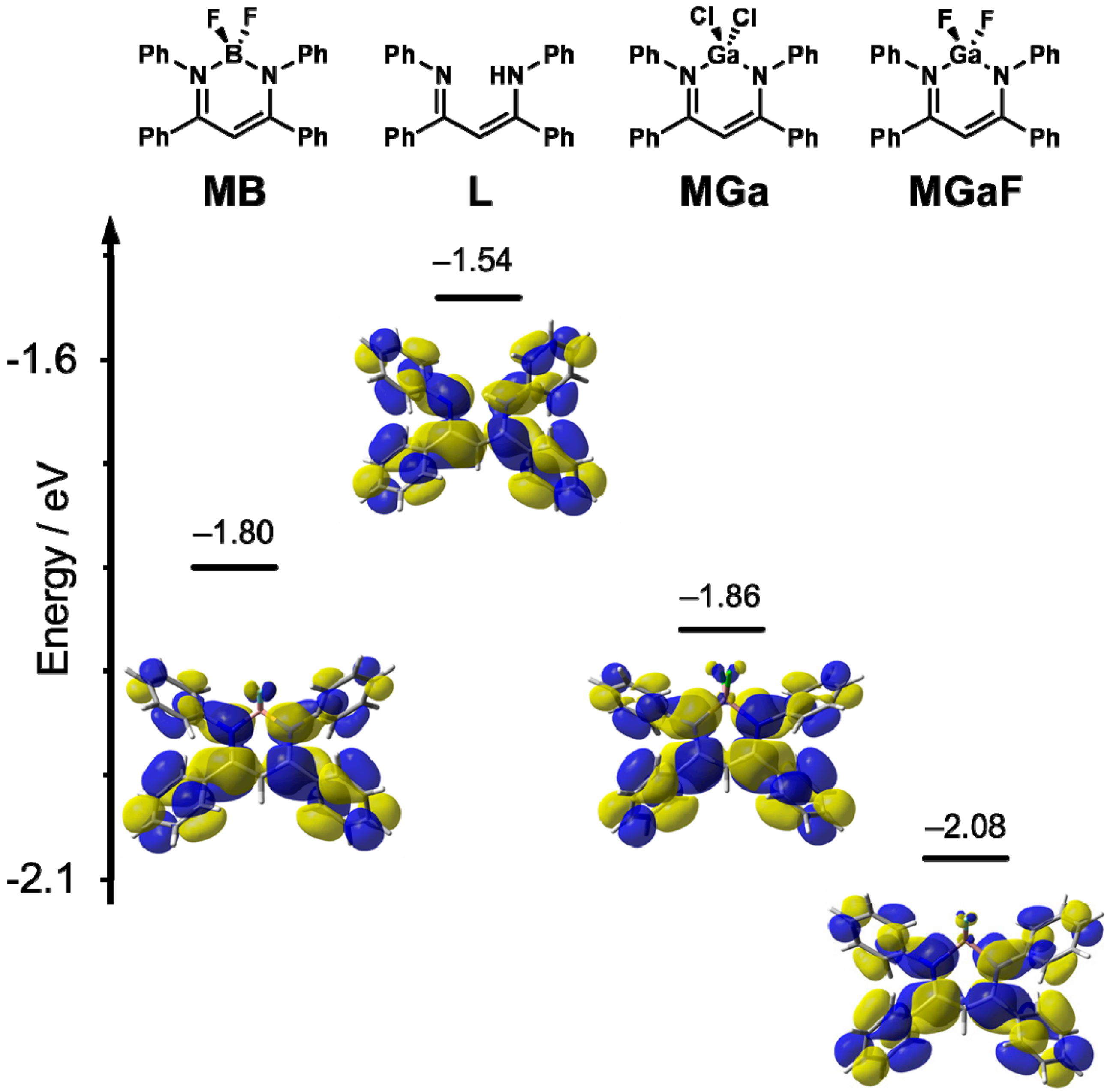

Figure S25. Energy diagrams and molecular orbital distributions of LUMOs for **L**, **MB**, **MGa** and **MGaF**, calculated by DFT calculations at a B3LYP level of theory with a 6-31G(d,p) basis set.

References

1. Yoshii, R.; Hirose, A.; Tanaka, K.; Chujo, Y. *J. Am. Chem. Soc.* **2014**, *136*, 18131–18139.

2. Yoshii, R.; Tanaka, K.; Chujo, Y. *Macromolecules* **2014**, *47*, 2268–2278.

3. (a) Valeur, B. Molecular Fluorescence: Principles and Applications, Wiley-VCH, Weinheim, 2002. (b) Kulkarni, P. A.; Wu, P. T.; Kwon, T. W.; Jenekhe, S. A. *J. Phys. Chem. B* **2005**, *109*, 19584–19594. (c) Filarowski, A.; Kluba, M.; Cieślik-Boczula, K.; Koll, A.; Kochel, A.; Pandey, L.; De Borggraeve, W. M.; Auweraer, M. V. d.; Catalán, J.; Boens, N. *Photochem. Photobiol. Sci.*, **2010**, *9*, 996–1008. (d) Pina, J.; de Melo, J. S.; Breusov, D.; Scherf, U. *Phys. Chem. Chem. Phys.* **2013**, *15*, 15204–15213.
